# Identification of microRNAs as potential markers of ovarian toxicity

**DOI:** 10.1002/jat.3583

**Published:** 2018-01-29

**Authors:** Hayley C. Furlong, Martin R. Stämpfli, Anne M. Gannon, Warren G. Foster

**Affiliations:** ^1^ Department of Obstetrics and Gynecology McMaster University Hamilton Ontario Canada; ^2^ Department of Pathology and Molecular Medicine McMaster University Hamilton Ontario Canada

**Keywords:** biomarkers, follicle, folliculogenesis, infertility, microRNA, ovarian toxicology cigarette smoke, ovary, toxicology

## Abstract

Exposure to environmental toxicants has been associated with ovarian dysfunction yet sensitive biomarkers of adverse effect are lacking. We previously demonstrated that cigarette smoke exposure induced decreased relative ovarian weight, increased follicle loss and granulosa cell autophagy in mice. We postulate that cigarette smoke exposure will induce changes in the epigenome that can be used to reveal potential sensitive biomarkers of ovarian toxicity. Therefore, we evaluated differences in expression of 940 microRNAs (miRNAs), environmentally responsive small non‐coding genes that regulate expression of genes at the post‐transcriptional level, in ovarian tissue from 8‐week‐old female C57BL/6 mice exposed to room air or cigarette smoke 5 days per week for 8 weeks. A total of 152 miRNAs were dysregulated in expression, 17 of which were examined with quantitative polymerase chain reaction analysis. Using an online miRNA database tool, complete lists of predicted miRNA gene targets were generated, 12 of which were measured for their expression levels with quantitative polymerase chain reaction. An online bioinformatics resource database, DAVID generated functional classification lists of the target genes and their associated biological pathways. Results of the present pilot study suggest that miR‐379, miR‐15b, miR‐691, miR‐872 and miR‐1897‐5p are potentially useful markers of ovarian toxicity and dysfunction. Examination of the expression pattern of the target mRNA for these miRNA species demonstrated that cigarette smoke exposure induced significant changes that affect mitogen‐activated protein kinase signaling pathways. We therefore suggest that miRNAs could serve as sensitive markers of ovarian toxicity and elucidate affected pathways.

## Abstract 2

We tested the hypothesis that miRNAs could serve as novel markers of ovarian toxicity. In a pilot study, we identified miRNAs that are highly expressed in the ovaries of mice exposed to whole‐body cigarette smoke that we previously demonstrated had decreased relative ovarian weight, increased follicle loss and granulosa cell autophagy. Our data suggest that cigarette smoke exposure dysregulates the expression of miRNAs in the mitogen‐activated protein kinase signaling pathway and thus could serppve as markers of ovarian dysfunction.

## 1 INTRODUCTION

MicroRNAs (miRNAs) are small non‐coding genetic material that regulate expression of genes at the post‐transcriptional level, either by degradation of mRNA or repression of translation (Lee, Feinbaum, & Ambros, 1993). Making up approximately 1–3% of the human genome, miRNAs are highly conserved across a number of different species (Bartel, 2004). miRNAs are involved in post‐transcriptional gene regulation that includes the splicing, editing, transport, storage turnover and translation of mRNA (Carletti & Christenson, 2009). They are capable of downregulating gene expression through complementary binding to the 3′‐UTR region of target genes (Ha & Kim, 2014) and have been shown to regulate a number of cellular processes. Dysregulation of miRNAs has been associated with disease and cancer (Gilabert‐Estelles et al., 2012). Thus, differences in miRNA expression are anticipated with disruption of folliculogenesis and dysregulation of ovarian function.

Recent studies have shown that small non‐coding segments of double‐stranded RNA are important in ovarian function. Specifically, the dicer gene codes for an enzyme that cleaves double‐stranded RNA (dsRNA) and pre‐miRNA into small interfering RNA and miRNA. Dicer is essential for the development of the female reproductive tract and for fertility in mice (Gonzalez & Behringer, 2009). The importance of miRNAs to the oocyte has been extensively investigated with the generation of *Dicer1*–/– knockout mice, which are infertile but display “histological normal” ovaries. Even though *Dicer1* may not be directly responsible for the development of the oocyte, studies have revealed that *Dicer1*–/– mice have misaligned spindles in their oocytes disrupting germ cell development (Tang et al., 2007). Moreover, miRNAs are the most abundant class of small RNAs in the ovary (Imbar & Eisenberg, 2014). The application of microarray, high‐throughput quantitative polymerase chain reaction (qPCR) and next‐generation sequencing technologies has revealed some of the most abundantly expressed known miRNAs in the ovary across a range of different species, some of which include *let‐7e*, *miR‐21*, *miR‐99a*, *miR‐125b*, *miR‐126*, *miR‐143*, *miR‐145* and *miR‐199b*.

In the ovary, follicular development (folliculogenesis) and recruitment are highly regulated processes driven by endocrine and paracrine hormone signaling. The role of miRNAs in folliculogenesis and recruitment has previously been reviewed (Baley & Li, 2012; Nothnick, 2012). The miRNAs that are most abundant in the oocyte include *miR‐30*, *miR‐16*, *let‐7* and *miR‐17‐92* families (Toloubeydokhti, Bukulmez, & Chegini, 2008). The expression of miRNAs in granulosa cells is believed to have a direct effect on folliculogenesis and ovarian steroidogenesis (Imbar & Eisenberg, 2014). Specifically, androgen receptors have been reported to enhance the expression of anti‐apoptotic *miR‐125b*, which in turn has a positive effect on follicle development and survival, and have been suggested as potential targets for enhancing fertility rates in women with compromised ovarian follicle growth (Sen et al., 2014). Additionally, ovulation requires *miR‐200b* expression in mice (Hasuwa, Ueda, Ikawa, & Okabe, 2013) and thus is essential for female fertility.

Although the role of miRNAs in folliculogenesis is clear, the consequence of environmental toxicant exposure on miRNA expression and ovarian dysfunction is unknown. Moreover, toxicant‐induced infertility is thought to be regulated through changes in miRNA expression (Hale, Keating, Yang, & Ross, 2015) but miRNA dysregulation in the ovaries has yet to be determined. The mechanisms by which environmental chemicals interact with and modify miRNA expression are not well understood. However, oxidative stress and inflammation pathways, both of which are implicated in a number of disease states (Hou, Wang, & Baccarelli, 2011) are thought to modify miRNA expression. We have recently shown that cigarette smoke (CS) exposure attenuates follicle development, decreases circulating estradiol concentrations and ultimately results in loss of primordial follicles (Gannon, Stämpfli, & Foster, 2013; Tuttle, Stämpfli, & Foster, 2009) via autophagy in mice (Furlong, Stämpfli, Gannon, & Foster, 2015; Gannon et al., 2013). We therefore hypothesize that the adverse effects of CS we have documented in the ovary will be accompanied by changes in miRNA expression, which could elucidate potentially useful markers of toxic insult and ovarian dysfunction. Therefore, to test this hypothesis, we used ovarian tissue from our earlier study (Furlong et al., 2015) employing a model we have shown to induce primordial follicle loss and autophagy, to investigate differences in miRNA expression and elucidate target pathways.

## 2 MATERIALS AND METHODS

### 2.1 Ethics

All animal work was conducted using protocols approved by the McMaster University Animal Research Ethics Board (AUP: 14‐07‐24) and was in accordance with the Canadian Council for Animal Care guidelines for the use of animals in research.

### 2.2 Research animals

**Figure 1:**
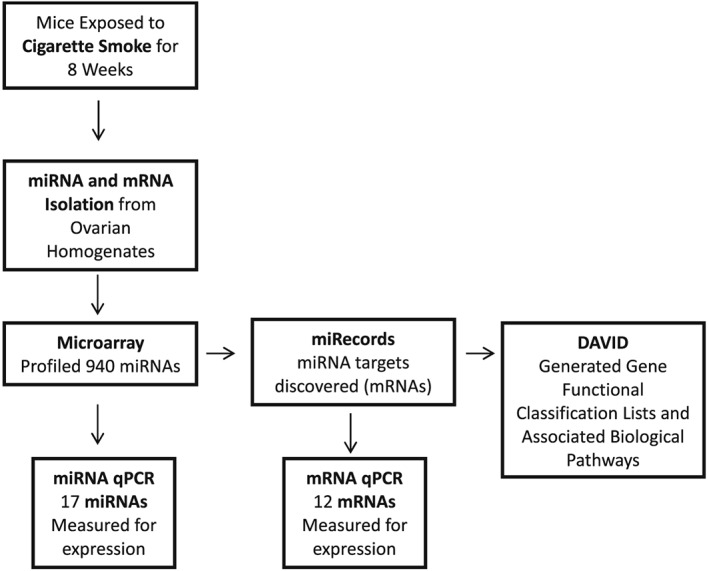
Overview of experimental methodology. Microarray = miRNA was measured for differences in expression of 940 of the most abundantly expressed miRNAs found in the mouse miRNome between control (*n* = 6) and cigarette smoke‐exposed (*n* = 6) ovaries of mice. miRNA qPCR = expression of miRNAs that were either statistically differentially expressed or had a fold change >2 in the miRNA array was examined. miRecords = miRNA target genes of the most dysregulated miRNAs (mostly elevated) were chosen for qPCR validation analysis. mRNA qPCR = miRNA target genes of the most dysregulated miRNAs (mostly elevated) were chosen for qPCR analysis. DAVID = functional classification lists of the mRNAs and their associated biological pathways were generated. qPCR, quantitative polymerase chain reaction

An overview of the experimental methodology is presented in Figure 1. Ovarian tissue used in this study was obtained from mice in which we previously documented CS‐induced ovarian toxicity (Furlong et al., 2015). Briefly, female C57BL/6 mice (8 weeks of age at the start of exposure) were obtained from Charles River Laboratories. Mice were maintained in polycarbonate cages at 22°C ± 2°C and 50% ± 10% relative humidity on a 12 hour light/12 hour dark photoperiod and were provided with normal rodent chow (LabDiet; PMI Nutrition International, St. Louis, MO) and tap water ad libitum throughout the experiment. Mice were exposed to CS twice daily, 5 days a week, for a total of 8 weeks using a whole‐body, mainstream smoke exposure system (SIU48; Promech Lab AB, Vintrie, Sweden) as previously described (Furlong et al., 2015) and equivalent to a pack a day in human exposure. Mice were killed at the end of the exposure period with carbon dioxide and exsanguinated. The ovaries were collected, immediately frozen and stored at –80°C for miRNA isolation and expression analyses.

### 2.3 microRNA/RNA isolation

Both miRNA and mRNA were isolated from ovarian homogenates using the miRNeasy mini kit (Qiagen, Toronto, ON, Canada) according to the manufacturer's instructions and quantified by spectrophotometric analysis (Nanodrop) and then reverse‐transcribed with either miScript II reverse transcription kit (Qiagen) or iScript kit (BioRad, Mississuaga, ON, Canada) and diluted with RNase‐free water and stored at –20°C until further use. Expression of miRNA/mRNA can be cycle dependent; however, in our previous work we have shown that housing of female mice in groups results in synchronization of estrous cycles with mice killed in metestrus to diestrus.

### 2.4 microRNA array

The reverse transcribed miRNA was measured for differences in expression of 940 of the most abundantly expressed miRNAs found in the mouse genome (miRNome) between control (*n* = 6) and CS‐exposed (*n* = 6) ovaries of mice using qPCR SYBR Green technology (Qiagen) and the miRNome miScript miRNA 384 well plate PCR Array kit (Qiagen catalog no. MIMM‐3216Z) and the Roche LightCycler 480 PCR System (Roche Diagnostics, Laval, QC, Canada). The complete list of miRNAs investigated can be found in Supporting information Table 1. We used six internal normalization controls/reference miRNAs (Snord‐61, ‐68, ‐72, ‐95, ‐96A and Rnu6‐2) in the array, all of which were unaltered in the array and thus were used for data analysis with the ΔΔ*C*_T_ method of relative quantification (http://pcrdataanalysis.sabiosciences.com). The fold change and *P* value results were included in all subsequent graphed data. Only reference genes that showed no statistically significant changes in expression between the control and CS groups were selected for analysis. A total of 152 miRNAs were differentially expressed in the CS‐exposed ovaries of mice.

### 2.5 Quantitative polymerase chain reaction expression of microRNAs from the array

A list of the miRNAs that were either statistically differentially expressed or had a fold change >2 in the miRNA array was generated (Supporting information Table 2a). The expression of these miRNAs (*let‐7e‐5p*, *miR‐101a‐3p*, *miR‐125b‐1‐3p*, *miR‐151‐5p*, *miR‐155‐3p*, *miR‐15b‐5p*, *miR‐188‐3p*, *miR‐1897‐3p*, *miR‐1a‐1‐5p*, *miR‐1b‐3p*, *miR‐208b‐5p*, *miR‐221‐5p*, *miR‐324‐5p*, *miR‐34b‐5p*, *miR‐379‐5*, *miR‐691 and miR‐872*) were subsequently examined using qPCR and miScript PCR SYBR Green kit Technology (Qiagen) and measured for their changes in expression between control (*n* = 8) and CS‐exposed (*n* = 8) ovaries of mice using murine‐specific commercial primers (Qiagen). The primers used are listed in Table 1. Unfortunately, some of the commercial miRNA primers employed failed to produce a signal (*miR‐1a‐1‐5p*, *miR‐324‐5p*, *miR‐155‐3p* and *miR‐1b‐3p (1‐2‐as‐3p‐6)*) and therefore were not included in the analysis. All samples were run in duplicate for both the miRNAs of interest and references genes. Relative quantification was performed (Schmittgen & Livak, 2008) and corrected for sample‐to‐sample differences with the reference genes generating normalized ratios of the miRNA/reference genes. In addition, a complete literature search was performed to reveal potential functions of the differentially expressed miRNAs.

**Table 1:** List of the commercial miRNA primers for quantitative polymerase chain reaction expression and mRNA expression in the ovary of mice exposed to cigarette smoke

miRNA	miRNA accession number	Sequences	Microarray fold‐change
*mmu‐let‐7e‐5p*	MIMAT0017016	5′‐CUAUACGGCCUCCUAGCUUUCC	3.35
*mmu‐miR‐101a‐3p*	MIMAT0000133	5′‐UACAGUACUGUGAUAACUGAA	3.26
*mmu‐miR‐125b‐1‐3p*	MIMAT0004669	5′‐ACGGGUUAGGCUCUUGGGAGCU	–25.08
*mmu‐miR‐151‐5p*	MIMAT0004536	5′‐UCGAGGAGCUCACAGUCUAGU	–5.18
*mmu‐miR‐155‐3p*	MIMAT0016993	5′‐CUCCUACCUGUUAGCAUUAAC	–3.05
*mmu‐miR‐15b‐5p*	MIMAT0000124	5′‐UAGCAGCACAUCAUGGUUUACA	–5.41
*mmu‐miR‐188‐3p*	MIMAT0004541	5′‐CUCCCACAUGCAGGGUUUGCA	–4.28
*mmu‐miR‐1897‐3p*	MIMAT0007865	5′‐UCAACUCGUUCUGUCCGGUGAG	14.22
*mmu‐miR‐1a‐1‐5p*	MIMAT0016979	5′‐ACAUACUUCUUUAUAUGCCCAUA	2.58
*mmu‐miR‐1b‐3p (1‐2‐as‐3p‐6)*	MIMAT0017326	5′‐UGGGUACAUAAAGAAGUAUGUGC	–3.84
*mmu‐miR‐208b‐5p*	MIMAT0017280	5′‐AAGCUUUUUGCUCGCGUUAUGU	3.66
*mmu‐miR‐221‐5p*	MIMAT0017060	5′‐ACCUGGCAUACAAUGUAGAUUUCUGU	2.16
*mmu‐miR‐324‐5p*	MIMAT0000555	5′‐CGCAUCCCCUAGGGCAUUGGUGU	2.39
*mmu‐miR‐34b‐5p*	MIMAT0000382	5′‐AGGCAGUGUAAUUAGCUGAUUGU	7.82
*mmu‐miR‐379‐5p*	MIMAT0000743	5′‐UGGUAGACUAUGGAACGUAGG	8.81
*mmu‐miR‐691*	MIMAT0003470	5′‐AUUCCUGAAGAGAGGCAGAAAA	–5.32
*mmu‐miR‐872‐5p*	MIMAT0004934	5′‐AAGGUUACUUGUUAGUUCAGG	–5.43

### 2.6 microRNA target prediction and target mRNA expression

The purpose of target prediction analysis was to reveal miRNA target genes (mRNA) that that may be dysregulated by the CS‐induced alterations in miRNA expression. The online miRNA database miRecords (http://c1.accurascience.com/miRecords) was selected as the most suitable for this study as it integrates 11 target prediction programs (DIANA‐microT, MicroInspector, miRanda, MirTarget2, miTarget, NBmiRTar, PicTar, PITA, RNA22, RNAhybrid and TargetScan) from which extensive lists of both predicted and validated putative miRNA target genes can be generated (Xiao et al., 2009). The complete list of miRNA gene targets is provided in Supporting information Table 2b. Only targets predicted by >4 of the 11 programs were included in this list. Using this information, miRNA target genes of the most dysregulated miRNAs (mostly elevated) were chosen for qPCR analysis and are highlighted in Supporting information Table 2b. The target genes were designed using the online Primer3 program as previously described (Furlong et al., 2015) and a list of the genes and their forward and reverse sequences are listed in Table 2. The target mRNA expression levels were quantified and normalized to the internal control gene, *Actb*.

**Table 2:** List of miRNA target genes (mRNA) used for quantitative polymerase chain reaction expression. There are no predicted or validated genes for *mir‐1897*

miRNA	Target gene (mRNA)	Gene forward primer	Gene reverse primer
*let‐7e*	*Tlr4*	ACCAAGCCTTTCAGGGAATTA	GATCAACCGATGGACGTGTA
*miR‐101a*	*Ptgs2/Cox‐2*	TTCCAAACCAGCAGACTCATAC	GCTCAGGTGTTGCACGTAGT
*miR‐101a*	*Dusp1 (Mkp‐1)*	CGGATGCAGCTCCTGTAGT	CCAAGGCGTCAAGCATATC
*miR‐15b*	*Esrrg*	TGGACTCGCCACCTCTCTAC	TGCTGGAGCAGTCATCATACA
*miR‐188*	*Atg9a*	ACAAGCGTGAGCTGACAGAGT	ACCAGGGCCACCATGTAGT
*miR‐34b*	*Vegf*	ACTGGACCCTGGCTTTACTG	GCAGTAGCTTCGCTGGTAGA
*miR‐34b*	*Myc*	CGACTCCGTACAGCCCTATT	TCTGCTGTTGCTGGTGATAGA
*miR‐34b*	*Creb1*	GCTCATTTCCAGCGATGAGTA	TGAACTGCACAGGAATGGTAGT
*miR‐379*	*Cdkn2aip*	CAAGTTTGCTTGTCGGAAGTTA	TGAAGCCACCAACTGCTACT
*miR‐691*	*Map3k7ip3*	ACCATCTCCTCGGGTGATAC	TGTTGCTCGGCCTACAGTAAT
*miR‐872*	*Mecp2*	GCCTGAGCCACAGAAGAGTATG	CCACCTAGCCTGCCTGTACT
*miR‐872*	*Anxa7*	CAGCCACCTGCACAGTCTTA	GAGCTTGTCCTCCAGGGTACT

### 2.7 Functional analysis (DAVID software)

Data from the miRecords prediction/validation list was extrapolated into the Database for Annotation Visualization Integrated Discovery (DAVID) software for functional analysis (https://david.ncifcrf.gov/). Pathways were identified from the Kyoto encyclopedia of genes and genomes (KEGG) database (Ogata et al., 1999).

### 2.8 Statistical analysis

Statistical analysis of the miRNA array data was performed through the SABiosciences online data analysis tool (http://pcrdataanalysis.sabiosciences.com), which calculates the fold up‐ or downregulation of miRNAs and compares the differences between control and CS groups with Student's *t*‐test, and *P* ≤ .05 was considered significant. Furthermore, miRNAs that had a fold‐change >2 were also considered potentially biologically relevant.

SigmaStat3.5 was used to assess statistical differences for the miRNA qPCR validation and mRNA qPCR analysis. Data were initially checked for normality by applying a D'Agostino and Pearson omnibus normality test, followed by either a *t*‐test (normally distributed data) or a Mann–Whitney (non‐normally distributed data) test. The qPCR results are presented as normalized mean ratios and ±SEM of the target/reference gene.

## 3 RESULTS

### 3.1 microRNA expression by polymerase chain reaction array

From the miRNA array, we found a total of 152 miRNAs that differed in expression between control ovarian tissue and CS ovarian tissues of mice. Of the miRNAs that were differentially expressed in the ovaries of CS‐exposed mice compared to controls, the expression of eight miRNAs (miR‐15b, ‐125b‐1‐3p, ‐151‐5p, ‐425‐3p, ‐691, ‐872‐5p, ‐1957a and ‐3474) differed from controls by fivefold or expression was significantly different (miR‐1b‐3p and miR‐155‐3p; *P* < .05) from the control group (Supporting information Table 2a). An additional 17 miRNAs were selected for further study by qPCR expression based on their biological relevance in reproductive tissues (*let‐7e‐5p*, *miR‐101a‐3p*, *miR‐1897‐3p*, *miR‐1a‐1‐5p*, *miR‐208b‐5p*, *miR‐221‐5p*, *miR‐324‐5p*, *miR‐34b‐5p*, *miR‐379‐5p*, *miR‐125b‐1‐3p*, *miR‐151‐5p*, *miR‐155‐3p*, *miR‐15b‐5p*, *mmu‐miR‐188‐3p*, *miR‐1b‐3p (1‐2‐as‐3p‐6)*, *miR‐691 and miR‐872*).

### 3.2 microRNA expression by quantitative polymerase chain reaction

**Figure 2:**
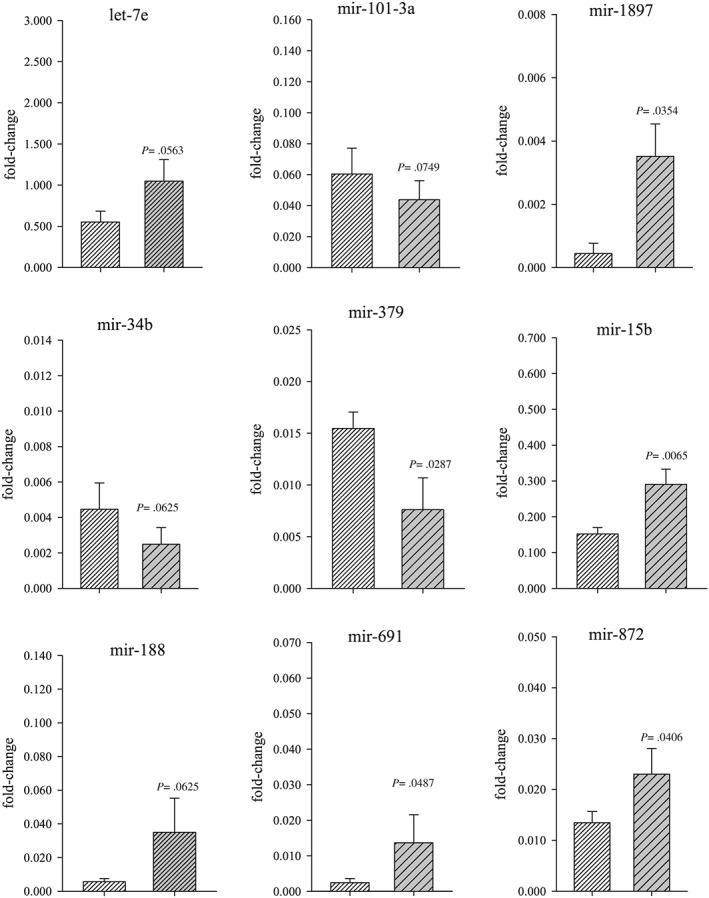
miRNA expression assessed by real‐time polymerase chain reaction. Changes in expression levels of each miRNA were quantified relative to the internal control genes: *Snord 61*, *‐68*, *‐72*, *‐95*, *‐96A* and *Rnu6‐2*. Lighter shaded bar represents the control group and the darker shaded bar represents the smoke exposed group. Quantitative polymerase chain reaction results are presented as normalized mean ratios and ±SEM of the target/reference gene

Based on the results of our PCR array data, the expression levels of the miRNAs listed above were measured with qPCR using commercially produced primers (Qiagen). In CS‐exposed ovarian tissue, *miR‐15b*, *miR‐691*, *miR‐872* and *miR‐1897‐5p* were significantly (*P* < .05) elevated in expression whereas only *miR‐379* was decreased in expression (Figure 2). Only primers that produced a single melting peak were included for analysis. Changes in expression of *miR‐125b‐1‐3p*, *miR‐151‐5p*, *miR‐155‐3p*, *miR‐1a‐1‐5p*, *miR‐1b‐3p*, *miR‐208b‐5p*, *miR‐221‐5p* and *miR‐324‐5p* had either insufficient fluorescence or changes in expression were not statistically significant and therefore not explored further.

### 3.3 microRNA target prediction and target mRNA expression

**Figure 3:**
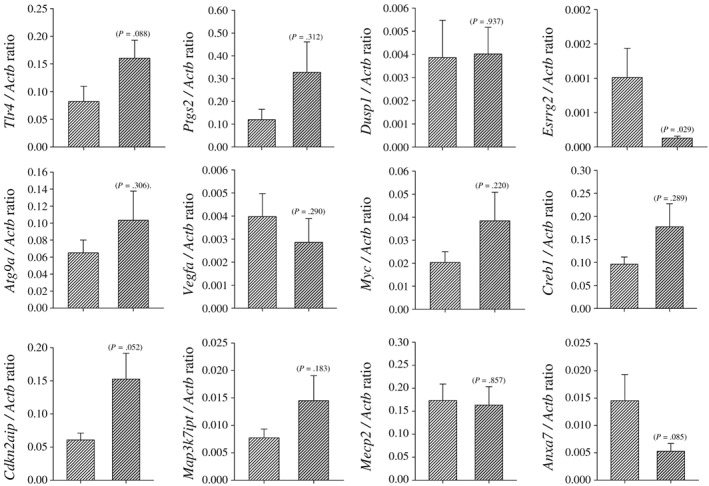
miRNA target gene expression was measured by qPCR. Target mRNA expression levels were quantified and normalized to the internal control gene, *Actb*. Lighter shaded bar represents the control group and darker shaded bar represents the smoke‐exposed group. Student's *t*‐test was investigated for statistically significant changes between the treatment groups, and the individual *P* values are recorded on each figure

A complete list of the putative miRNA targets (mRNAs) of the miRNAs that differed in the CS‐exposed ovarian tissue was generated with miRecords (Supporting information Table 2b). The interaction of miRNA and their target mRNAs (interactome) that were further investigated with qPCR analysis is presented as follows in Table 2: ***miRNA***/*target gene*; ***let‐7e***/*Tlr4*, ***miR101a***/*Ptgs2*, ***miR101a***/*Dusp1*, ***miR‐15b***/*Esrrg* **, *miR‐188***/*Atg9a*, ***miR‐34b***/*Myc*, ***miR‐34b***/*Creb1*, ***miR‐34b***/*Vegf*, ***miR‐379***/*Cdkn2aip*, ***miR‐691***/*Map3k7ip3*, ***miR‐872***/*Mecp2* and ***miR‐872***/*Anxa7*. Ratios of miRNA target gene expression relative to the reference genes were compared (Figure 3). Of the target genes examined only the expression of *Esrrg2* was significantly decreased (*P* = .029) whereas *cdkn2aip*, *Anxa7* and *Tlr4* expression was non‐significantly elevated (*P* = .052, .085 and .088, respectively) in ovarian tissue from CS‐exposed mice compared to controls. No differences in the expression of other target genes between CS‐exposed and control mice were detectable.

### 3.4 Functional analysis

To elucidate biological meaning from the dysregulated expression of miRNA targets and reveal potential signaling pathways implicated in the CS‐exposed ovaries of mice, functional analysis was performed. miRNA target genes were grouped according to function and ranked from the highest to lowest enrichment groups. Gene functional classification results are presented in Supporting information Table 3. Functional annotation clustering, functional annotation chart and table are presented in Supporting information Table 4. The mitogen‐activated protein kinase (MAPK) signaling pathway appeared as the top enriched pathway of the target genes of the dysregulated miRNAs and is shown in Table 3.

**Table 3:** Predicted pathways targeted by miRNAs expressed in cigarette smoke exposed female mouse ovaries. DAVID and KEGG pathway analysis suggest that the miRNAs were generally involved in MAPK signaling

Pathway	*P* Value	% of genes in pathway that are present
MAPK signaling pathway	1.123E‐02	3.9
Transcriptional misregulation in cancer	3.581E‐02	2.8
MicroRNAs in cancer	5.574E‐02	3.4

MAPK, mitogen‐activated protein kinase.

## 4 DISCUSSION

In the present study, we found that of 152 miRNAs whose expression was dysregulated in response to CS the expression of eight miRNAs differed from the control group by greater than fivefold and the expression of two miRNAs was significantly different from the control group. Dysregulation of expression was confirmed by target qPCR and are generally associated with cell cycle regulation, cell death, proliferation and inflammation. Examination of the predicted dysregulated pathways identified using DAVID on the list of putative target mRNAs, demonstrated that CS exposure induced significant changes that affect MAPK signaling.

Analysis of miRNA expression by qPCR showed that *miR‐15b*, *miR‐1897‐5p*, *miR‐691* and *miR‐872* were significantly upregulated whereas *miR‐379* was significantly downregulated. We suggest that CS‐induced dysregulation of these miRNAs are important in the development of ovarian toxicity. For example, *miR‐15b* expression was significantly upregulated using qPCR in this pilot study and is known to target genes involved in cell cycle regulation and cell death. Specifically, *miR‐15b* is upregulated in head and neck squamous cell carcinoma cell lines (Nakanishi et al., 2014) and in human trophoblast and endothelial cells in the placenta abnormal expression of *miR‐15* was associated with improper placentation (Yang et al., 2016). Increased expression has also been linked to an influx of mitochondrial‐associated reactive oxygen species and negative regulation of mitochondrial sirtuin (SIRT4), indicative of cellular senescence (Lang et al., 2016). Our result further revealed that its target gene, estrogen‐related receptor gamma (*Esrrg*), was significantly downregulated, suggesting that some estrogen‐regulated targets are suppressed by CS exposure; findings consistent with the anti‐estrogenic effects of CS exposure.

In the current study, *miR‐1897‐5p* expression was significantly upregulated in ovarian homogenates from mice exposed to CS compared to controls. Elevated levels of *miR‐1897‐5p* expression have been associated with increased inflammation and apoptotic cell death (Bellinger et al., 2014). Expression of *miR‐691* was also found to be upregulated in the current study. The *miR‐691* target gene, MAPK 7‐interacting protein 3 (*Map3k7ip3*) responds to proinflammatory cytokines through the nuclear factor B signal transduction (*Nfkb*) pathway, which was measured for its expression; however, it was found to be unchanged. *miR‐872* expression was significantly upregulated by qPCR and is thought to be directly regulated by insulin (Chang et al., 2011). Target genes *Mecp2* showed no significant change in expression and *Anxa7* expression was non‐significantly downregulated. One of the challenges associated with target identification is that binding of miRNAs to their target genes might not result in alteration of the level of target gene expression (Toloubeydokhti et al., 2008) and therefore may be indicative of the resulting discrepancies between the miRNA/target gene expression. The remaining unchanged miRNA expression profiles may have regulatory roles for maintaining normal physiological function and therefore may not be altered in expression.

qPCR analysis in the present study revealed that only *miR‐379* expression was significantly downregulated by CS exposure. Differentially expressed levels of the miR‐379 have also been found in smokers (Guled et al., 2009). Moreover, downregulated levels of miR‐379 have been associated with the development of human cancers (Laddha et al., 2013), in particular, breast cancer (Khan et al., 2013), and cigarette smoking has been linked with an increased risk of developing breast cancer (Gram et al., 2005; Land, Liu, Wickerham, Costantino, & Ganz, 2014). Expression of the *miR‐379* target gene, cyclin‐dependent kinase inhibitor 2A (*Cdkn2aip*), was borderline significantly upregulated (*P =* .052). *Cdkn2a* (or *p16*), the official name of *Cdkn2aip*, is capable of inducing cell cycle arrest and apoptosis in a p53‐dependent manner by preventing the activation of cyclin complexes (Stott et al., 1998). This is important with respect to our study because it is suggestive of an induction of cellular damage followed by arrested apoptosis and an alternative cellular death pathway and consistent with our previous studies demonstrating CS exposure induced granulosa cell autophagy in the murine ovary (Furlong et al., 2015; Gannon et al., 2013).

The expression pattern for several miRNAs, *let‐7e*, *miR‐101a*, *miR‐34b* and *miR‐188*, were marginally significant. As we used whole ovary homogenates, which may dilute expression patterns by inclusion of non‐target cells, and thus bias our results toward the null hypothesis. Of these miRNA, *let*‐7e is particularly important because it is thought to repress mTOR signaling and induce autophagy (Dubinsky et al., 2014), which is harmonious with our previous findings of autophagy in the ovaries of CS‐exposed mice (Furlong et al., 2015; Gannon et al., 2013). Its target gene, toll‐like receptor 4 (*Tlr4*), was non‐significantly upregulated (*P* = .088) in the CS‐exposed ovary. *Tlr4* has an important role in the innate immune response through the recognition of pathogens (Lester & Li, 2014). Specifically, *Tlr4* is a lipopolysaccharide ligand that modulates autophagy through enhancing autophagosome formation in monocytes and macrophages coupled with activation of nuclear factor‐kappaB and MAPKs (Agrawal et al., 2015). Furthermore, *Tlr4* has been associated with activated cellular death pathways in ovarian granulosa cells of obese women, due to the increased concentrations of oxidized low‐density lipoprotein and subsequent oxidative stress (Schube et al., 2014).

Some studies have reported a number of miRNAs as potential novel markers of damage following CS exposure but these markers could not be confirmed by the results of the present pilot study. For example, clinical investigations measuring the role of miRNAs as mediators of CS in utero show significant downregulation of *miR‐16*, *miR‐21* and *miR‐146a* in the placental tissues of mothers who smoke (Maccani et al., 2010), and results in overproduction of their complementary mRNAs. Similarly, increased *miR‐223* expression was correlated with increased maternal cotinine levels in maternal and cord blood and decreased levels of T‐regulatory cells, which is suggestive of a relationship between CS and immune responses mediated through regulatory miRNAs (Herberth et al., 2014).

Gene functional analysis of the upregulated miRNA target genes revealed a potential role for MAPK signaling. The MAPK pathway is involved in cell proliferation, differentiation, cellular death/survival and motility and consequently, dysregulation of the MAPK pathway has been linked with disease (Rauch, Rukhlenko, Kolch, & Kholodenko, 2016). In reproduction, side stream CS has been known to be equally as damaging as active smoking (Neal, Hughes, Holloway, & Foster, 2005), and a study investigating the effects of side stream CS exposure in epithelial cells revealed activation of MAPK signaling suggestive of a CS target (Low, Liang, & Fu, 2007). Subsequently, the authors showed that treatment with the aryl hydrocarbon receptor antagonist and anti‐oxidant compound resveratrol, the activation of MAPK could be mitigated, protecting the cells from side stream CS damage. In mice, MAPK has been suggested as a potential target for the treatment of chronic obstructive pulmonary disease, resulting from CS exposure and the same results have been shown in subsequent studies (Low et al., 2007; Marumo et al., 2014). Taken together these results suggest that the MAPK signaling pathway may play a role in CS‐induced tissue damage.

The present study has a number of strengths. Specifically, we used tissue collected from ovaries in mice exposed to CS, at concentrations representative of women who smoke a pack per day, and in which we have previously documented ovarian follicle loss (Gannon et al., 2013; Tuttle et al., 2009) and granulosa cell autophagy (Furlong et al., 2015). A limitation of the present study is the use of whole ovarian homogenates, which precludes determining follicle compartments and cell types specifically targeted by CS exposure. We suggest that the ability to detect toxicant‐induced changes in ovarian function would be enhanced in future studies by use of laser capture microdissection.

In conclusion, we identified several miRNAs involved in CS‐induced ovarian dysregulation. Predictive pathway analysis suggested dysregulation of the MAPK signaling pathway. Taken together the results of the present study lay the foundation for further investigations into the use of miRNA targets as markers of ovarian insult and dysregulation.

## AUTHORS’ ROLES

AG was responsible for the exposure of mice to cigarette smoke system belonging to MS. HF was responsible for preparation of tissues and performed the experiments and analysis. HF was also responsible for the preparation of the manuscript under the guidance and direction of WF. Both HF and WF designed the overall experimental plan.

## CONFLICT OF INTEREST

The authors did not report any conflict of interest.

## Supporting information

Supporting Information

Supporting Information

Supporting Information

Supporting Information

Supporting Information
