# Supplementary material for: Identification of microRNAs as potential markers of ovarian toxicity
Source: J Appl Toxicol. 2018 Jan 29;38(5):744–52. doi: 10.1002/jat.3583 (PMC5901046; doi:10.1002/jat.3583)
Supplement: Supplementary file 4 — Supporting Information [file JAT-38-744-s004.docx]

|  | **Mouse miRNome Plate 1 MIMM-301** |  | **Mouse miRNome Plate 1 MIMM-302** |  | **Mouse miRNome Plate 1 MIMM-303** |  |  |  |  |
| --- | --- | --- | --- | --- | --- | --- | --- | --- | --- |
|  | **Position** | **miRNA ID** | **Position** | **miRNA ID** | **Position** | **miRNA ID** |  |  |  |
|  | A01 | mmu-miR-183-5p | A01 | mmu-miR-1898 | A01 | mmu-miR-1894-5p |  |  |  |
|  | A02 | mmu-miR-877-5p | A02 | mmu-miR-1899 | A02 | mmu-miR-1929-5p |  |  |  |
|  | A03 | mmu-miR-15b-5p | A03 | mmu-miR-18a-3p | A03 | mmu-miR-194-2-3p |  |  |  |
|  | A04 | mmu-miR-9-3p | A04 | mmu-miR-190a-5p | A04 | mmu-miR-653-3p |  |  |  |
|  | A05 | mmu-miR-199a-5p | A05 | mmu-miR-1900 | A05 | mmu-miR-3110-5p |  |  |  |
|  | A06 | mmu-miR-369-5p | A06 | mmu-miR-1901 | A06 | mmu-miR-326-5p |  |  |  |
|  | A07 | mmu-miR-101a-3p | A07 | mmu-miR-1902 | A07 | mmu-miR-1930-3p |  |  |  |
|  | A08 | mmu-miR-324-5p | A08 | mmu-miR-1903 | A08 | mmu-miR-3080-5p |  |  |  |
|  | A09 | mmu-miR-292-3p | A09 | mmu-miR-1904 | A09 | mmu-miR-32-3p |  |  |  |
|  | A10 | mmu-miR-676-3p | A10 | mmu-miR-1905 | A10 | mmu-miR-873a-3p |  |  |  |
|  | A11 | mmu-miR-194-5p | A11 | mmu-miR-1906 | A11 | mmu-miR-379-5p |  |  |  |
|  | A12 | mmu-miR-449a-5p | A12 | mmu-miR-191-3p | A12 | mmu-miR-3088-3p |  |  |  |
|  | A13 | mmu-miR-712-5p | A13 | mmu-miR-1928 | A13 | mmu-miR-200c-3p |  |  |  |
|  | A14 | mmu-miR-34b-5p | A14 | mmu-miR-193a-5p | A14 | mmu-miR-34a-3p |  |  |  |
|  | A15 | mmu-miR-470-5p | A15 | mmu-miR-1935 | A15 | mmu-miR-143-5p |  |  |  |
|  | A16 | mmu-miR-184-3p | A16 | mmu-miR-1938 | A16 | mmu-miR-3070b-3p |  |  |  |
|  | A17 | mmu-miR-193b-3p | A17 | mmu-miR-1942 | A17 | mmu-miR-1251-5p |  |  |  |
|  | A18 | mmu-miR-431-5p | A18 | mmu-miR-1946a | A18 | mmu-miR-3059-5p |  |  |  |
|  | A19 | mmu-miR-483-3p | A19 | mmu-miR-1947-5p | A19 | mmu-miR-1843a-3p |  |  |  |
|  | A20 | mmu-miR-449c-5p | A20 | mmu-miR-1948-3p | A20 | mmu-miR-1948-5p |  |  |  |
|  | A21 | mmu-miR-149-5p | A21 | mmu-miR-1949 | A21 | mmu-miR-377-5p |  |  |  |
|  | A22 | mmu-miR-215-5p | A22 | mmu-miR-1950 | A22 | mmu-miR-432 |  |  |  |
|  | A23 | mmu-miR-191-5p | A23 | mmu-miR-1954 | A23 | mmu-miR-3065-3p |  |  |  |
|  | A24 | mmu-miR-331-5p | A24 | mmu-miR-1957a | A24 | mmu-miR-1952 |  |  |  |
|  | B01 | mmu-miR-200b-5p | B01 | mmu-miR-1962 | B01 | mmu-miR-208b-5p |  |  |  |
|  | B02 | mmu-miR-92a-3p | B02 | mmu-miR-1983 | B02 | mmu-miR-696 |  |  |  |
|  | B03 | mmu-miR-770-3p | B03 | mmu-miR-199b-5p | B03 | mmu-miR-135a-2-3p |  |  |  |
|  | B04 | mmu-miR-326-3p | B04 | mmu-miR-200a-5p | B04 | mmu-miR-3082-3p |  |  |  |
|  | B05 | mmu-miR-210-3p | B05 | mmu-miR-203-5p | B05 | mmu-miR-3108-3p |  |  |  |
|  | B06 | mmu-miR-216a-5p | B06 | mmu-miR-204-5p | B06 | mmu-miR-182-3p |  |  |  |
|  | B07 | mmu-miR-125a-5p | B07 | mmu-miR-207 | B07 | mmu-miR-3079-5p |  |  |  |
|  | B08 | mmu-miR-126a-5p | B08 | mmu-miR-20a-3p | B08 | mmu-miR-18b-3p |  |  |  |
|  | B09 | mmu-miR-376c-3p | B09 | mmu-miR-20b-3p | B09 | mmu-miR-871-5p |  |  |  |
|  | B10 | mmu-miR-466g | B10 | mmu-miR-218-1-3p | B10 | mmu-miR-19b-1-5p |  |  |  |
|  | B11 | mmu-miR-138-5p | B11 | mmu-miR-218-2-3p | B11 | mmu-miR-1956 |  |  |  |
|  | B12 | mmu-miR-346-5p | B12 | mmu-miR-22-5p | B12 | mmu-miR-701-3p |  |  |  |
|  | B13 | mmu-miR-18a-5p | B13 | mmu-miR-24-1-5p | B13 | mmu-miR-1264-5p |  |  |  |
|  | B14 | mmu-miR-665-3p | B14 | mmu-miR-24-2-5p | B14 | mmu-miR-652-5p |  |  |  |
|  | B15 | mmu-miR-345-3p | B15 | mmu-miR-26a-5p | B15 | mmu-miR-493-5p |  |  |  |
|  | B16 | mmu-miR-290a-5p | B16 | mmu-miR-26b-3p | B16 | mmu-miR-1a-2-5p |  |  |  |
|  | B17 | mmu-let-7f-5p | B17 | mmu-miR-27a-5p | B17 | mmu-miR-132-5p |  |  |  |
|  | B18 | mmu-miR-134-5p | B18 | mmu-miR-28a-3p | B18 | mmu-miR-3072-3p |  |  |  |
|  | B19 | mmu-miR-216b-5p | B19 | mmu-miR-293-3p | B19 | mmu-miR-1947-3p |  |  |  |
|  | B20 | mmu-miR-362-3p | B20 | mmu-miR-293-5p | B20 | mmu-miR-1197-3p |  |  |  |
|  | B21 | mmu-miR-434-5p | B21 | mmu-miR-294-3p | B21 | mmu-miR-27b-5p |  |  |  |
|  | B22 | mmu-miR-342-3p | B22 | mmu-miR-295-5p | B22 | mmu-miR-3074-5p |  |  |  |
|  | B23 | mmu-miR-125b-5p | B23 | mmu-miR-297b-5p | B23 | mmu-miR-673-3p |  |  |  |
|  | B24 | mmu-miR-874-3p | B24 | mmu-miR-297c-5p | B24 | mmu-miR-669f-3p |  |  |  |
|  | C01 | mmu-miR-672-5p | C01 | mmu-miR-299a-5p // mmu-miR-299b-5p | C01 | mmu-miR-3472 |  |  |  |
|  | C02 | mmu-miR-382-5p | C02 | mmu-miR-29a-5p | C02 | mmu-miR-3093-3p |  |  |  |
|  | C03 | mmu-miR-872-5p | C03 | mmu-miR-29c-5p | C03 | mmu-miR-196a-2-3p |  |  |  |
|  | C04 | mmu-miR-484 | C04 | mmu-miR-300-5p | C04 | mmu-miR-3083-3p |  |  |  |
|  | C05 | mmu-miR-688 | C05 | mmu-miR-302b-5p | C05 | mmu-miR-1298-3p |  |  |  |
|  | C06 | mmu-miR-122-5p | C06 | mmu-miR-30a-3p | C06 | mmu-miR-490-5p |  |  |  |
|  | C07 | mmu-miR-710 | C07 | mmu-miR-30b-3p | C07 | mmu-miR-450a-1-3p |  |  |  |
|  | C08 | mmu-miR-455-3p | C08 | mmu-miR-30c-1-3p | C08 | mmu-miR-760-5p |  |  |  |
|  | C09 | mmu-miR-880-3p | C09 | mmu-miR-30c-2-3p | C09 | mmu-miR-101b-5p |  |  |  |
|  | C10 | mmu-miR-485-5p | C10 | mmu-miR-30e-3p | C10 | mmu-miR-669b-3p |  |  |  |
|  | C11 | mmu-miR-146a-5p | C11 | mmu-miR-31-3p | C11 | mmu-miR-136-3p |  |  |  |
|  | C12 | mmu-miR-489-3p | C12 | mmu-miR-325-5p | C12 | mmu-miR-3102-3p |  |  |  |
|  | C13 | mmu-miR-21a-5p | C13 | mmu-miR-33-3p | C13 | mmu-miR-297a-5* |  |  |  |
|  | C14 | mmu-miR-196b-5p | C14 | mmu-miR-330-3p | C14 | mmu-miR-1965 |  |  |  |
|  | C15 | mmu-miR-137-3p | C15 | mmu-miR-339-3p | C15 | mmu-miR-99a-3p |  |  |  |
|  | C16 | mmu-miR-377-3p | C16 | mmu-miR-340-5p | C16 | mmu-miR-1839-5p |  |  |  |
|  | C17 | mmu-miR-654-5p | C17 | mmu-miR-365-3p | C17 | mmu-miR-92a-2-5p |  |  |  |
|  | C18 | mmu-miR-677-5p | C18 | mmu-miR-374b-3p | C18 | mmu-miR-667-5p |  |  |  |
|  | C19 | mmu-miR-671-3p | C19 | mmu-miR-376a-5p | C19 | mmu-miR-3076-5p |  |  |  |
|  | C20 | mmu-miR-466d-3p | C20 | mmu-miR-376b-3p | C20 | mmu-miR-383-3p |  |  |  |
|  | C21 | mmu-miR-224-5p | C21 | mmu-miR-376c-5p | C21 | mmu-miR-466h-5p |  |  |  |
|  | C22 | mmu-miR-434-3p | C22 | mmu-miR-378a-5p | C22 | mmu-miR-3057-5p |  |  |  |
|  | C23 | mmu-miR-375-3p | C23 | mmu-miR-382-3p | C23 | mmu-miR-3082-5p |  |  |  |
|  | C24 | mmu-miR-302b-3p | C24 | mmu-miR-410-3p | C24 | mmu-miR-18b-5p |  |  |  |
|  | D01 | mmu-miR-652-3p | D01 | mmu-miR-411-3p | D01 | mmu-miR-1955-5p |  |  |  |
|  | D02 | mmu-miR-330-5p | D02 | mmu-miR-425-3p | D02 | mmu-miR-134-3p |  |  |  |
|  | D03 | mmu-miR-495-3p | D03 | mmu-miR-433-5p | D03 | mmu-miR-466l-5p |  |  |  |
|  | D04 | mmu-miR-667-3p | D04 | mmu-miR-450b-5p | D04 | mmu-miR-431-3p |  |  |  |
|  | D05 | mmu-miR-341-3p | D05 | mmu-miR-455-5p | D05 | mmu-miR-3112-5p |  |  |  |
|  | D06 | mmu-miR-541-5p | D06 | mmu-miR-463-5p | D06 | mmu-miR-379-3p |  |  |  |
|  | D07 | mmu-miR-30d-5p | D07 | mmu-miR-466j | D07 | mmu-miR-219a-1-3p |  |  |  |
|  | D08 | mmu-miR-582-3p | D08 | mmu-miR-466k | D08 | mmu-miR-129-1-3p |  |  |  |
|  | D09 | mmu-let-7a-5p | D09 | mmu-miR-467b-5p | D09 | mmu-miR-1198-5p |  |  |  |
|  | D10 | mmu-miR-376a-3p | D10 | mmu-miR-467f | D10 | mmu-miR-1193-5p |  |  |  |
|  | D11 | mmu-miR-34b-3p | D11 | mmu-miR-467h | D11 | mmu-miR-7b-3p |  |  |  |
|  | D12 | mmu-miR-21a-3p | D12 | mmu-miR-470-3p | D12 | mmu-miR-153-5p |  |  |  |
|  | D13 | mmu-miR-487b-3p | D13 | mmu-miR-485-3p | D13 | mmu-miR-3071-3p |  |  |  |
|  | D14 | mmu-miR-22-3p | D14 | mmu-miR-488-5p | D14 | mmu-miR-664-3p |  |  |  |
|  | D15 | mmu-miR-721 | D15 | mmu-miR-493-3p | D15 | mmu-miR-200b-3p |  |  |  |
|  | D16 | mmu-miR-504-5p | D16 | mmu-miR-503-3p | D16 | mmu-miR-1907 |  |  |  |
|  | D17 | mmu-miR-200a-3p | D17 | mmu-miR-540-3p | D17 | mmu-miR-3475-3p |  |  |  |
|  | D18 | mmu-miR-378a-3p | D18 | mmu-miR-551b-3p | D18 | mmu-miR-3075-3p |  |  |  |
|  | D19 | mmu-miR-29c-3p | D19 | mmu-miR-568 | D19 | mmu-miR-471-3p |  |  |  |
|  | D20 | mmu-miR-294-5p | D20 | mmu-miR-598-3p | D20 | mmu-miR-344e-5p // mmu-miR-344h-5p |  |  |  |
|  | D21 | mmu-miR-467b-3p | D21 | mmu-miR-615-5p | D21 | mmu-miR-547-5p |  |  |  |
|  | D22 | mmu-miR-340-3p | D22 | mmu-miR-666-5p | D22 | mmu-miR-208a-5p |  |  |  |
|  | D23 | mmu-miR-290a-3p | D23 | mmu-miR-669e-5p | D23 | mmu-let-7e-3p |  |  |  |
|  | D24 | mmu-miR-380-5p | D24 | mmu-miR-669g | D24 | mmu-miR-367-3p |  |  |  |
|  | E01 | mmu-miR-744-5p | E01 | mmu-miR-669h-3p | E01 | mmu-miR-598-5p |  |  |  |
|  | E02 | mmu-miR-500-3p | E02 | mmu-miR-669h-5p | E02 | mmu-miR-195a-3p |  |  |  |
|  | E03 | mmu-miR-155-5p | E03 | mmu-miR-669i | E03 | mmu-miR-1224-5p |  |  |  |
|  | E04 | mmu-miR-101b-3p | E04 | mmu-miR-669j | E04 | mmu-miR-320-5p |  |  |  |
|  | E05 | mmu-miR-875-3p | E05 | mmu-miR-669k-3p | E05 | mmu-miR-3064-5p |  |  |  |
|  | E06 | mmu-miR-29a-3p | E06 | mmu-miR-674-3p | E06 | mmu-miR-3068-5p |  |  |  |
|  | E07 | mmu-miR-302c-5p | E07 | mmu-miR-675-3p | E07 | mmu-miR-1930-5p |  |  |  |
|  | E08 | mmu-miR-130a-3p | E08 | mmu-miR-676-5p | E08 | mmu-miR-212-3p |  |  |  |
|  | E09 | mmu-miR-882 | E09 | mmu-miR-682 | E09 | mmu-miR-100-3p |  |  |  |
|  | E10 | mmu-miR-542-5p | E10 | mmu-miR-693-3p | E10 | mmu-miR-874-5p |  |  |  |
|  | E11 | mmu-miR-203-3p | E11 | mmu-miR-693-5p | E11 | mmu-miR-128-2-5p |  |  |  |
|  | E12 | mmu-miR-27a-3p | E12 | mmu-miR-694 | E12 | mmu-miR-543-5p |  |  |  |
|  | E13 | mmu-miR-301b-3p | E13 | mmu-miR-695 | E13 | mmu-miR-3104-3p |  |  |  |
|  | E14 | mmu-miR-331-3p | E14 | mmu-miR-697 | E14 | mmu-miR-181c-3p |  |  |  |
|  | E15 | mmu-miR-34c-5p | E15 | mmu-miR-708-3p | E15 | mmu-miR-544-5p |  |  |  |
|  | E16 | mmu-miR-218-5p | E16 | mmu-miR-709 | E16 | mmu-miR-381-5p |  |  |  |
|  | E17 | mmu-miR-302d-3p | E17 | mmu-miR-741-3p | E17 | mmu-miR-505-3p |  |  |  |
|  | E18 | mmu-miR-187-3p | E18 | mmu-miR-742-5p | E18 | mmu-miR-138-1-3p |  |  |  |
|  | E19 | mmu-miR-188-5p | E19 | mmu-miR-743b-3p | E19 | mmu-miR-3060-5p |  |  |  |
|  | E20 | mmu-miR-139-5p | E20 | mmu-miR-744-3p | E20 | mmu-miR-3088-5p |  |  |  |
|  | E21 | mmu-miR-19a-3p | E21 | mmu-miR-872-3p | E21 | mmu-miR-1a-1-5p |  |  |  |
|  | E22 | mmu-miR-343 | E22 | mmu-miR-877-3p | E22 | mmu-miR-450a-2-3p |  |  |  |
|  | E23 | mmu-miR-295-3p | E23 | mmu-miR-878-5p | E23 | mmu-let-7f-2-3p |  |  |  |
|  | E24 | mmu-miR-181c-5p | E24 | mmu-miR-879-5p | E24 | mmu-miR-3091-5p |  |  |  |
|  | F01 | mmu-miR-23b-3p | F01 | mmu-miR-879-3p | F01 | mmu-miR-367-5p |  |  |  |
|  | F02 | mmu-miR-532-5p | F02 | mmu-miR-93-3p | F02 | mmu-miR-351-3p |  |  |  |
|  | F03 | mmu-miR-876-5p | F03 | mmu-miR-99b-3p | F03 | mmu-miR-1927 |  |  |  |
|  | F04 | mmu-miR-133b-3p | F04 | mmu-miR-1946b | F04 | mmu-miR-106a-3p |  |  |  |
|  | F05 | mmu-miR-192-5p | F05 | mmu-miR-881-3p | F05 | mmu-miR-23a-5p |  |  |  |
|  | F06 | mmu-miR-883a-3p | F06 | mmu-miR-297a-5p | F06 | mmu-let-7a-2-3p |  |  |  |
|  | F07 | mmu-miR-883b-3p | F07 | mmu-miR-30d-3p | F07 | mmu-miR-873a-5p |  |  |  |
|  | F08 | mmu-miR-770-5p | F08 | mmu-miR-3084-3p | F08 | mmu-miR-871-3p |  |  |  |
|  | F09 | mmu-miR-883b-5p | F09 | mmu-miR-344b-5p | F09 | mmu-miR-222-5p |  |  |  |
|  | F10 | mmu-miR-323-5p | F10 | mmu-miR-700-5p | F10 | mmu-miR-181b-2-3p |  |  |  |
|  | F11 | mmu-miR-322-5p | F11 | mmu-miR-511-5p | F11 | mmu-miR-540-5p |  |  |  |
|  | F12 | mmu-miR-222-3p | F12 | mmu-miR-125b-2-3p | F12 | mmu-miR-452-5p |  |  |  |
|  | F13 | mmu-miR-590-5p | F13 | mmu-miR-92a-1-5p | F13 | mmu-miR-3101-3p |  |  |  |
|  | F14 | mmu-miR-425-5p | F14 | mmu-miR-322-3p | F14 | mmu-miR-16-2-3p |  |  |  |
|  | F15 | mmu-miR-144-3p | F15 | mmu-miR-758-5p | F15 | mmu-let-7f-1-3p |  |  |  |
|  | F16 | mmu-miR-804 | F16 | mmu-miR-23b-5p | F16 | mmu-miR-350-5p |  |  |  |
|  | F17 | mmu-miR-719 | F17 | mmu-miR-3062-3p | F17 | mmu-miR-466n-3p |  |  |  |
|  | F18 | mmu-miR-759 | F18 | mmu-miR-450a-5p | F18 | mmu-miR-3090-5p |  |  |  |
|  | F19 | mmu-miR-345-5p | F19 | mmu-miR-471-5p | F19 | mmu-miR-1964-5p |  |  |  |
|  | F20 | mmu-miR-30b-5p | F20 | mmu-miR-500-5p | F20 | mmu-miR-344c-3p |  |  |  |
|  | F21 | mmu-miR-25-3p | F21 | mmu-miR-302c-3p | F21 | mmu-miR-2136 |  |  |  |
|  | F22 | mmu-miR-875-5p | F22 | mmu-miR-361-3p | F22 | mmu-miR-129-2-3p |  |  |  |
|  | F23 | mmu-miR-433-3p | F23 | mmu-miR-135b-3p | F23 | mmu-miR-3092-5p |  |  |  |
|  | F24 | mmu-miR-509-5p | F24 | mmu-miR-374c-3p | F24 | mmu-miR-204-3p |  |  |  |
|  | G01 | mmu-miR-883a-5p | G01 | mmu-miR-190b-3p | G01 | mmu-miR-206-3p |  |  |  |
|  | G02 | mmu-miR-31-5p | G02 | mmu-miR-1249-3p | G02 | mmu-miR-449a-3p |  |  |  |
|  | G03 | mmu-miR-201-5p | G03 | mmu-miR-344d-1-5p | G03 | mmu-miR-3086-5p |  |  |  |
|  | G04 | mmu-miR-142-3p | G04 | mmu-miR-196b-3p | G04 | mmu-miR-1933-3p |  |  |  |
|  | G05 | mmu-miR-686 | G05 | mmu-miR-511-3p | G05 | mmu-miR-211-3p |  |  |  |
|  | G06 | mmu-miR-291a-5p | G06 | mmu-miR-3099-5p | G06 | mmu-miR-3079-3p |  |  |  |
|  | G07 | mmu-miR-296-5p | G07 | mmu-miR-3077-3p | G07 | mmu-miR-3109-3p |  |  |  |
|  | G08 | mmu-miR-361-5p | G08 | mmu-miR-429-5p | G08 | mmu-miR-143-3p |  |  |  |
|  | G09 | mmu-miR-351-5p | G09 | mmu-miR-3100-3p | G09 | mmu-miR-1249-5p |  |  |  |
|  | G10 | mmu-miR-103-3p | G10 | mmu-miR-3087-3p | G10 | mmu-miR-669m-3p |  |  |  |
|  | G11 | mmu-miR-148b-3p | G11 | mmu-miR-3102-5p.2-5p | G11 | mmu-miR-219a-5p |  |  |  |
|  | G12 | mmu-let-7g-5p | G12 | mmu-miR-3059-3p | G12 | mmu-miR-3099-3p |  |  |  |
|  | G13 | mmu-miR-409-5p | G13 | mmu-miR-672-3p | G13 | mmu-miR-3074-1-5p |  |  |  |
|  | G14 | mmu-miR-190b-5p | G14 | mmu-miR-201-3p | G14 | mmu-miR-1298-5p |  |  |  |
|  | G15 | mmu-miR-16-5p | G15 | mmu-miR-3063-5p | G15 | mmu-miR-1951 |  |  |  |
|  | G16 | mmu-miR-409-3p | G16 | mmu-miR-802-3p | G16 | mmu-miR-1943-5p |  |  |  |
|  | G17 | mmu-miR-876-3p | G17 | mmu-miR-496a-5p | G17 | mmu-miR-3089-3p |  |  |  |
|  | G18 | mmu-miR-126a-3p | G18 | mmu-miR-3071-5p | G18 | mmu-miR-499-3p |  |  |  |
|  | G19 | mmu-miR-320-3p | G19 | mmu-miR-3103-3p | G19 | mmu-miR-194-1-3p |  |  |  |
|  | G20 | mmu-miR-381-3p | G20 | mmu-miR-3085-5p | G20 | mmu-miR-1940 |  |  |  |
|  | G21 | mmu-miR-182-5p | G21 | mmu-miR-1945 | G21 | mmu-miR-483-5p |  |  |  |
|  | G22 | mmu-miR-590-3p | G22 | mmu-miR-448-5p | G22 | mmu-miR-467g |  |  |  |
|  | G23 | mmu-miR-106a-5p | G23 | mmu-miR-3069-5p | G23 | mmu-miR-668-3p |  |  |  |
|  | G24 | mmu-miR-532-3p | G24 | mmu-miR-3057-3p | G24 | mmu-miR-1941-5p |  |  |  |
|  | H01 | mmu-miR-142-5p | H01 | mmu-miR-670-5p | H01 | mmu-miR-221-5p |  |  |  |
|  | H02 | mmu-miR-186-5p | H02 | mmu-miR-495-5p | H02 | mmu-miR-466o-3p |  |  |  |
|  | H03 | mmu-miR-107-3p | H03 | mmu-miR-539-5p | H03 | mmu-miR-147-5p |  |  |  |
|  | H04 | mmu-miR-29b-3p | H04 | mmu-miR-181d-3p | H04 | mmu-miR-301a-5p |  |  |  |
|  | H05 | mmu-miR-329-3p | H05 | mmu-miR-187-5p | H05 | mmu-miR-34c-3p |  |  |  |
|  | H06 | mmu-miR-92b-3p | H06 | mmu-miR-216b-3p | H06 | mmu-miR-96-3p |  |  |  |
|  | H07 | mmu-miR-291b-3p | H07 | mmu-miR-3076-3p | H07 | mmu-miR-3109-5p |  |  |  |
|  | H08 | mmu-miR-202-3p | H08 | mmu-miR-219a-2-3p | H08 | mmu-miR-1961 |  |  |  |
|  | H09 | mmu-miR-675-5p | H09 | mmu-miR-3095-3p | H09 | mmu-miR-3098-5p |  |  |  |
|  | H10 | mmu-miR-146b-5p | H10 | mmu-miR-3066-3p | H10 | mmu-miR-107-5p |  |  |  |
|  | H11 | mmu-miR-411-5p | H11 | mmu-miR-509-3p | H11 | mmu-miR-767 |  |  |  |
|  | H12 | mmu-miR-878-3p | H12 | mmu-miR-148b-5p | H12 | mmu-miR-29b-2-5p |  |  |  |
|  | H13 | mmu-miR-466f-5p | H13 | mmu-miR-3094-3p | H13 | mmu-miR-669c-5p |  |  |  |
|  | H14 | mmu-miR-802-5p | H14 | mmu-miR-490-3p | H14 | mmu-miR-103-1-5p |  |  |  |
|  | H15 | mmu-let-7b-5p | H15 | mmu-miR-3092-3p | H15 | mmu-miR-344b-3p |  |  |  |
|  | H16 | mmu-miR-302a-5p | H16 | mmu-miR-592-3p | H16 | mmu-miR-669a-5p // mmu-miR-669p-5p |  |  |  |
|  | H17 | mmu-miR-98-5p | H17 | mmu-miR-505-5p | H17 | mmu-miR-3107-3p // mmu-miR-486-3p |  |  |  |
|  | H18 | mmu-miR-714 | H18 | mmu-miR-1264-3p | H18 | mmu-miR-486-5p // mmu-miR-3107-5p |  |  |  |
|  | H19 | mmu-miR-762 | H19 | mmu-miR-181b-1-3p | H19 | mmu-miR-467a-3p // mmu-miR-467d-3p |  |  |  |
|  | H20 | mmu-miR-327 | H20 | mmu-miR-344g-5p | H20 | mmu-miR-466a-5p // mmu-miR-466p-5p |  |  |  |
|  | H21 | mmu-miR-19b-3p | H21 | mmu-miR-3112-3p | H21 | mmu-miR-374b-5p // mmu-miR-374c-5p |  |  |  |
|  | H22 | mmu-miR-376b-5p | H22 | mmu-miR-196a-1-3p | H22 | mmu-miR-3070a-5p // mmu-miR-3070b-5p |  |  |  |
|  | H23 | mmu-miR-328-3p | H23 | mmu-miR-713 | H23 | mmu-miR-199a-3p // mmu-miR-199b-3p |  |  |  |
|  | H24 | mmu-miR-338-5p | H24 | mmu-miR-3069-3p | H24 | mmu-miR-1982-3p // mmu-miR-1982.2-3p |  |  |  |
|  | I01 | mmu-miR-196a-5p | I01 | mmu-miR-764-5p | I01 | mmu-let-7a-1-3p // mmu-let-7c-2-3p |  |  |  |
|  | I02 | mmu-miR-380-3p | I02 | mmu-miR-3087-5p | I02 | mmu-miR-28a-5p // mmu-miR-28c |  |  |  |
|  | I03 | mmu-miR-701-5p | I03 | mmu-miR-3072-5p | I03 | mmu-miR-3096a-5p // mmu-miR-3096b-5p |  |  |  |
|  | I04 | mmu-miR-350-3p | I04 | mmu-miR-369-3p | I04 | mmu-miR-3096a-3p // mmu-miR-3096b-3p |  |  |  |
|  | I05 | mmu-miR-154-3p | I05 | mmu-miR-1936 | I05 | Empty |  |  |  |
|  | I06 | mmu-miR-467c-5p | I06 | mmu-miR-135a-1-3p | I06 | Empty |  |  |  |
|  | I07 | mmu-miR-125a-3p | I07 | mmu-miR-1251-3p | I07 | Empty |  |  |  |
|  | I08 | mmu-let-7c-5p | I08 | mmu-miR-3066-5p | I08 | Empty |  |  |  |
|  | I09 | mmu-miR-881-5p | I09 | mmu-miR-1981-5p | I09 | Empty |  |  |  |
|  | I10 | mmu-miR-674-5p | I10 | mmu-miR-1247-5p | I10 | Empty |  |  |  |
|  | I11 | mmu-miR-654-3p | I11 | mmu-miR-146a-3p | I11 | Empty |  |  |  |
|  | I12 | mmu-miR-546 | I12 | mmu-miR-223-5p | I12 | Empty |  |  |  |
|  | I13 | mmu-miR-100-5p | I13 | mmu-miR-205-3p | I13 | Empty |  |  |  |
|  | I14 | mmu-miR-20b-5p | I14 | mmu-miR-2183 | I14 | Empty |  |  |  |
|  | I15 | mmu-miR-362-5p | I15 | mmu-miR-1b-5p | I15 | Empty |  |  |  |
|  | I16 | mmu-miR-133a-3p | I16 | mmu-miR-133b-5p | I16 | Empty |  |  |  |
|  | I17 | mmu-miR-700-3p | I17 | mmu-miR-26a-1-3p | I17 | Empty |  |  |  |
|  | I18 | mmu-miR-615-3p | I18 | mmu-miR-363-3p | I18 | Empty |  |  |  |
|  | I19 | mmu-miR-467e-5p | I19 | mmu-miR-677-3p | I19 | Empty |  |  |  |
|  | I20 | mmu-miR-708-5p | I20 | mmu-miR-1933-5p | I20 | Empty |  |  |  |
|  | I21 | mmu-miR-217-5p | I21 | mmu-miR-370-5p | I21 | Empty |  |  |  |
|  | I22 | mmu-miR-301a-3p | I22 | mmu-miR-1981-3p | I22 | Empty |  |  |  |
|  | I23 | mmu-miR-140-3p | I23 | mmu-miR-3064-3p | I23 | Empty |  |  |  |
|  | I24 | mmu-miR-503-5p | I24 | mmu-miR-3105-3p | I24 | Empty |  |  |  |
|  | J01 | mmu-let-7d-5p | J01 | mmu-miR-679-3p | J01 | Empty |  |  |  |
|  | J02 | mmu-miR-335-3p | J02 | mmu-miR-3062-5p | J02 | Empty |  |  |  |
|  | J03 | mmu-miR-26b-5p | J03 | mmu-miR-3105-5p | J03 | Empty |  |  |  |
|  | J04 | mmu-miR-542-3p | J04 | mmu-miR-3083-5p | J04 | Empty |  |  |  |
|  | J05 | mmu-miR-19a-5p | J05 | mmu-miR-3098-3p | J05 | Empty |  |  |  |
|  | J06 | mmu-miR-135b-5p | J06 | mmu-miR-3061-3p | J06 | Empty |  |  |  |
|  | J07 | mmu-miR-720 | J07 | mmu-miR-539-3p | J07 | Empty |  |  |  |
|  | J08 | mmu-miR-195a-5p | J08 | mmu-miR-486-3p | J08 | Empty |  |  |  |
|  | J09 | mmu-miR-711 | J09 | mmu-miR-3101-5p | J09 | Empty |  |  |  |
|  | J10 | mmu-miR-34a-5p | J10 | mmu-miR-215-3p | J10 | Empty |  |  |  |
|  | J11 | mmu-miR-130b-3p | J11 | mmu-miR-136-5p | J11 | Empty |  |  |  |
|  | J12 | mmu-let-7e-5p | J12 | mmu-miR-329-5p | J12 | Empty |  |  |  |
|  | J13 | mmu-miR-681 | J13 | mmu-miR-541-3p | J13 | Empty |  |  |  |
|  | J14 | mmu-miR-188-3p | J14 | mmu-miR-760-3p | J14 | Empty |  |  |  |
|  | J15 | mmu-miR-32-5p | J15 | mmu-miR-1955-3p | J15 | Empty |  |  |  |
|  | J16 | mmu-let-7i-5p | J16 | mmu-miR-224-3p | J16 | Empty |  |  |  |
|  | J17 | mmu-miR-208b-3p | J17 | mmu-miR-302d-5p | J17 | Empty |  |  |  |
|  | J18 | mmu-miR-135a-5p | J18 | mmu-miR-1968-5p | J18 | Empty |  |  |  |
|  | J19 | mmu-miR-707 | J19 | mmu-miR-1953 | J19 | Empty |  |  |  |
|  | J20 | mmu-miR-298-5p | J20 | mmu-miR-302a-3p | J20 | Empty |  |  |  |
|  | J21 | mmu-miR-666-3p | J21 | mmu-miR-3058-3p | J21 | Empty |  |  |  |
|  | J22 | mmu-miR-181b-5p | J22 | mmu-miR-3094-5p | J22 | Empty |  |  |  |
|  | J23 | mmu-miR-466c-5p | J23 | mmu-miR-463-3p | J23 | Empty |  |  |  |
|  | J24 | mmu-miR-384-3p | J24 | mmu-miR-344g-3p | J24 | Empty |  |  |  |
|  | K01 | mmu-miR-200c-5p | K01 | mmu-miR-3091-3p | K01 | Empty |  |  |  |
|  | K02 | mmu-miR-140-5p | K02 | mmu-miR-92b-5p | K02 | Empty |  |  |  |
|  | K03 | mmu-miR-7a-5p | K03 | mmu-miR-3093-5p | K03 | Empty |  |  |  |
|  | K04 | mmu-miR-185-5p | K04 | mmu-miR-1b-3p | K04 | Empty |  |  |  |
|  | K05 | mmu-miR-496a-3p | K05 | mmu-miR-466l-3p | K05 | Empty |  |  |  |
|  | K06 | mmu-miR-129-5p | K06 | mmu-miR-592-5p | K06 | Empty |  |  |  |
|  | K07 | mmu-miR-758-3p | K07 | mmu-miR-138-2-3p | K07 | Empty |  |  |  |
|  | K08 | mmu-miR-128-3p | K08 | mmu-miR-3097-3p | K08 | Empty |  |  |  |
|  | K09 | mmu-miR-30e-5p | K09 | mmu-miR-298-3p | K09 | Empty |  |  |  |
|  | K10 | mmu-miR-339-5p | K10 | mmu-miR-3067-5p | K10 | Empty |  |  |  |
|  | K11 | mmu-miR-337-3p | K11 | mmu-miR-3080-3p | K11 | Empty |  |  |  |
|  | K12 | mmu-miR-342-5p | K12 | mmu-miR-3106-3p | K12 | Empty |  |  |  |
|  | K13 | mmu-miR-20a-5p | K13 | mmu-miR-599 | K13 | Empty |  |  |  |
|  | K14 | mmu-miR-429-3p | K14 | mmu-miR-206-5p | K14 | Empty |  |  |  |
|  | K15 | mmu-miR-141-3p | K15 | mmu-miR-344c-5p | K15 | Empty |  |  |  |
|  | K16 | mmu-miR-7b-5p | K16 | mmu-miR-1197-5p | K16 | Empty |  |  |  |
|  | K17 | mmu-miR-93-5p | K17 | mmu-miR-127-3p | K17 | Empty |  |  |  |
|  | K18 | mmu-miR-30c-5p | K18 | mmu-miR-421-5p | K18 | Empty |  |  |  |
|  | K19 | mmu-miR-543-3p | K19 | mmu-miR-665-5p | K19 | Empty |  |  |  |
|  | K20 | mmu-miR-10a-5p | K20 | mmu-miR-1968-3p | K20 | Empty |  |  |  |
|  | K21 | mmu-miR-449b | K21 | mmu-miR-3063-3p | K21 | Empty |  |  |  |
|  | K22 | mmu-miR-338-3p | K22 | mmu-miR-193b-5p | K22 | Empty |  |  |  |
|  | K23 | mmu-miR-214-3p | K23 | mmu-miR-210-5p | K23 | Empty |  |  |  |
|  | K24 | mmu-miR-574-3p | K24 | mmu-miR-3060-3p | K24 | Empty |  |  |  |
|  | L01 | mmu-miR-335-5p | L01 | mmu-miR-3084-5p | L01 | Empty |  |  |  |
|  | L02 | mmu-miR-453 | L02 | mmu-miR-491-5p | L02 | Empty |  |  |  |
|  | L03 | mmu-miR-291a-3p | L03 | mmu-miR-3078-5p | L03 | Empty |  |  |  |
|  | L04 | mmu-miR-211-5p | L04 | mmu-miR-29b-1-5p | L04 | Empty |  |  |  |
|  | L05 | mmu-miR-684 | L05 | mmu-miR-19b-2-5p | L05 | Empty |  |  |  |
|  | L06 | mmu-miR-27b-3p | L06 | mmu-miR-494-5p | L06 | Empty |  |  |  |
|  | L07 | mmu-miR-205-5p | L07 | mmu-miR-344d-3p | L07 | Empty |  |  |  |
|  | L08 | mmu-miR-683 | L08 | mmu-miR-410-5p | L08 | Empty |  |  |  |
|  | L09 | mmu-miR-337-5p | L09 | mmu-miR-344d-2-5p | L09 | Empty |  |  |  |
|  | L10 | mmu-miR-742-3p | L10 | mmu-miR-128-1-5p | L10 | Empty |  |  |  |
|  | L11 | mmu-miR-384-5p | L11 | mmu-miR-1188-3p | L11 | Empty |  |  |  |
|  | L12 | mmu-miR-325-3p | L12 | mmu-miR-25-5p | L12 | Empty |  |  |  |
|  | L13 | mmu-miR-501-5p | L13 | mmu-miR-1247-3p | L13 | Empty |  |  |  |
|  | L14 | mmu-miR-324-3p | L14 | mmu-miR-468-3p | L14 | Empty |  |  |  |
|  | L15 | mmu-miR-423-3p | L15 | mmu-miR-190a-3p | L15 | Empty |  |  |  |
|  | L16 | mmu-miR-451a | L16 | mmu-miR-214-5p | L16 | Empty |  |  |  |
|  | L17 | mmu-miR-132-3p | L17 | mmu-miR-1969 | L17 | Empty |  |  |  |
|  | L18 | mmu-miR-669b-5p | L18 | mmu-miR-1943-3p | L18 | Empty |  |  |  |
|  | L19 | mmu-miR-153-3p | L19 | mmu-miR-3086-3p | L19 | Empty |  |  |  |
|  | L20 | mmu-miR-96-5p | L20 | mmu-miR-3471 | L20 | Empty |  |  |  |
|  | L21 | mmu-miR-1a-3p | L21 | mmu-miR-217-3p | L21 | Empty |  |  |  |
|  | L22 | mmu-miR-10b-5p | L22 | mmu-miR-1970 | L22 | Empty |  |  |  |
|  | L23 | mmu-miR-653-5p | L23 | mmu-miR-301b-5p | L23 | Empty |  |  |  |
|  | L24 | mmu-miR-383-5p | L24 | mmu-miR-2137 | L24 | Empty |  |  |  |
|  | M01 | mmu-miR-300-3p | M01 | mmu-miR-363-5p | M01 | Empty |  |  |  |
|  | M02 | mmu-miR-9-5p | M02 | mmu-miR-375-5p | M02 | Empty |  |  |  |
|  | M03 | mmu-miR-743b-5p | M03 | mmu-miR-664-5p | M03 | Empty |  |  |  |
|  | M04 | mmu-miR-421-3p | M04 | mmu-miR-1941-3p | M04 | Empty |  |  |  |
|  | M05 | mmu-miR-148a-3p | M05 | mmu-miR-7a-2-3p | M05 | Empty |  |  |  |
|  | M06 | mmu-miR-181d-5p | M06 | mmu-miR-412-5p | M06 | Empty |  |  |  |
|  | M07 | mmu-miR-223-3p | M07 | mmu-miR-3070a-3p | M07 | Empty |  |  |  |
|  | M08 | mmu-miR-763 | M08 | mmu-miR-669o-5p | M08 | Empty |  |  |  |
|  | M09 | mmu-miR-702-3p | M09 | mmu-miR-743a-5p | M09 | Empty |  |  |  |
|  | M10 | mmu-miR-488-3p | M10 | mmu-miR-344f-3p | M10 | Empty |  |  |  |
|  | M11 | mmu-miR-15a-5p | M11 | mmu-miR-1934-5p | M11 | Empty |  |  |  |
|  | M12 | mmu-miR-24-3p | M12 | mmu-miR-551b-5p | M12 | Empty |  |  |  |
|  | M13 | mmu-miR-124-3p | M13 | mmu-miR-144-5p | M13 | Empty |  |  |  |
|  | M14 | mmu-miR-127-5p | M14 | mmu-miR-154-5p | M14 | Empty |  |  |  |
|  | M15 | mmu-miR-450b-3p | M15 | mmu-miR-3095-5p | M15 | Empty |  |  |  |
|  | M16 | mmu-miR-221-3p | M16 | mmu-miR-3075-5p | M16 | Empty |  |  |  |
|  | M17 | mmu-miR-202-5p | M17 | mmu-miR-504-3p | M17 | Empty |  |  |  |
|  | M18 | mmu-miR-678 | M18 | mmu-miR-344d-3-5p | M18 | Empty |  |  |  |
|  | M19 | mmu-miR-193a-3p | M19 | mmu-miR-1971 | M19 | Empty |  |  |  |
|  | M20 | mmu-miR-99a-5p | M20 | mmu-miR-1967 | M20 | Empty |  |  |  |
|  | M21 | mmu-miR-547-3p | M21 | mmu-miR-669k-5p | M21 | Empty |  |  |  |
|  | M22 | mmu-miR-151-3p | M22 | mmu-miR-16-1-3p | M22 | Empty |  |  |  |
|  | M23 | mmu-miR-33-5p | M23 | mmu-miR-491-3p | M23 | Empty |  |  |  |
|  | M24 | mmu-miR-497-5p | M24 | mmu-miR-1931 | M24 | Empty |  |  |  |
|  | N01 | mmu-miR-23a-3p | N01 | mmu-miR-344f-5p | N01 | Empty |  |  |  |
|  | N02 | mmu-miR-582-5p | N02 | mmu-miR-1306-3p | N02 | Empty |  |  |  |
|  | N03 | mmu-miR-761 | N03 | mmu-miR-691 | N03 | Empty |  |  |  |
|  | N04 | mmu-miR-465a-5p | N04 | mmu-miR-487b-5p | N04 | Empty |  |  |  |
|  | N05 | mmu-miR-687 | N05 | mmu-miR-717 | N05 | Empty |  |  |  |
|  | N06 | mmu-miR-106b-5p | N06 | mmu-miR-1224-3p | N06 | Empty |  |  |  |
|  | N07 | mmu-miR-152-3p | N07 | mmu-miR-216a-3p | N07 | Empty |  |  |  |
|  | N08 | mmu-miR-299a-3p | N08 | mmu-miR-1964-3p | N08 | Empty |  |  |  |
|  | N09 | mmu-miR-704 | N09 | mmu-miR-1894-3p | N09 | Empty |  |  |  |
|  | N10 | mmu-miR-17-5p | N10 | mmu-miR-670-3p | N10 | Empty |  |  |  |
|  | N11 | mmu-miR-30a-5p | N11 | mmu-miR-1960 | N11 | Empty |  |  |  |
|  | N12 | mmu-miR-764-3p | N12 | mmu-miR-3102-5p | N12 | Empty |  |  |  |
|  | N13 | mmu-miR-147-3p | N13 | mmu-miR-3108-5p | N13 | Empty |  |  |  |
|  | N14 | mmu-miR-150-5p | N14 | mmu-miR-1963 | N14 | Empty |  |  |  |
|  | N15 | mmu-miR-698-3p | N15 | mmu-miR-465b-5p | N15 | Empty |  |  |  |
|  | N16 | mmu-miR-296-3p | N16 | mmu-miR-365-1-5p | N16 | Empty |  |  |  |
|  | N17 | mmu-miR-743a-3p | N17 | mmu-miR-10a-3p | N17 | Empty |  |  |  |
|  | N18 | mmu-miR-151-5p | N18 | mmu-miR-130a-5p | N18 | Empty |  |  |  |
|  | N19 | mmu-miR-501-3p | N19 | mmu-miR-412-3p | N19 | Empty |  |  |  |
|  | N20 | mmu-miR-423-5p | N20 | mmu-miR-192-3p | N20 | Empty |  |  |  |
|  | N21 | mmu-miR-105 | N21 | mmu-miR-3068-3p | N21 | Empty |  |  |  |
|  | N22 | mmu-miR-124-5p | N22 | mmu-miR-208a-3p | N22 | Empty |  |  |  |
|  | N23 | mmu-let-7b-3p | N23 | mmu-miR-344e-3p | N23 | Empty |  |  |  |
|  | N24 | mmu-let-7c-1-3p | N24 | mmu-miR-155-3p | N24 | Empty |  |  |  |
|  | O01 | mmu-let-7d-3p | O01 | mmu-miR-3110-3p | O01 | Empty |  |  |  |
|  | O02 | mmu-let-7g-3p | O02 | mmu-miR-3097-5p | O02 | Empty |  |  |  |
|  | O03 | mmu-let-7i-3p | O03 | mmu-miR-465c-5p | O03 | Empty |  |  |  |
|  | O04 | mmu-miR-101a-5p | O04 | mmu-miR-1958 | O04 | Empty |  |  |  |
|  | O05 | mmu-miR-106b-3p | O05 | mmu-miR-122-3p | O05 | Empty |  |  |  |
|  | O06 | mmu-miR-10b-3p | O06 | mmu-miR-448-3p | O06 | Empty |  |  |  |
|  | O07 | mmu-miR-1187 | O07 | mmu-miR-212-5p | O07 | Empty |  |  |  |
|  | O08 | mmu-miR-1188-5p | O08 | mmu-miR-3065-5p | O08 | Empty |  |  |  |
|  | O09 | mmu-miR-1190 | O09 | mmu-miR-499-5p | O09 | Empty |  |  |  |
|  | O10 | mmu-miR-1191 | O10 | mmu-miR-679-5p | O10 | Empty |  |  |  |
|  | O11 | mmu-miR-1192 | O11 | mmu-miR-3090-3p | O11 | Empty |  |  |  |
|  | O12 | mmu-miR-1194 | O12 | mmu-miR-3061-5p | O12 | Empty |  |  |  |
|  | O13 | mmu-miR-1195 | O13 | mmu-miR-669e-3p | O13 | Empty |  |  |  |
|  | O14 | mmu-miR-1196-5p | O14 | mmu-miR-668-5p | O14 | Empty |  |  |  |
|  | O15 | mmu-miR-1199-5p | O15 | mmu-miR-3078-3p | O15 | Empty |  |  |  |
|  | O16 | mmu-miR-130b-5p | O16 | mmu-miR-1198-3p | O16 | Empty |  |  |  |
|  | O17 | mmu-miR-133a-5p | O17 | mmu-miR-328-5p | O17 | Empty |  |  |  |
|  | O18 | mmu-miR-141-5p | O18 | mmu-miR-1912-3p | O18 | Empty |  |  |  |
|  | O19 | mmu-miR-145a-5p | O19 | mmu-miR-26a-2-3p | O19 | Empty |  |  |  |
|  | O20 | mmu-miR-145a-3p | O20 | mmu-miR-1193-3p | O20 | Empty |  |  |  |
|  | O21 | mmu-miR-146b-3p | O21 | mmu-miR-452-3p | O21 | Empty |  |  |  |
|  | O22 | mmu-miR-148a-5p | O22 | mmu-miR-3073a-3p | O22 | Empty |  |  |  |
|  | O23 | mmu-miR-15a-3p | O23 | mmu-miR-7a-1-3p | O23 | Empty |  |  |  |
|  | O24 | mmu-miR-15b-3p | O24 | mmu-miR-152-5p | O24 | Empty |  |  |  |
|  | P01 | mmu-miR-17-3p | P01 | mmu-miR-544-3p | P01 | Empty |  |  |  |
|  | P02 | mmu-miR-181a-5p | P02 | mmu-miR-3106-5p | P02 | Empty |  |  |  |
|  | P03 | mmu-miR-181a-1-3p | P03 | mmu-miR-98-3p | P03 | Empty |  |  |  |
|  | P04 | mmu-miR-181a-2-3p | P04 | mmu-miR-1932 | P04 | Empty |  |  |  |
|  | P05 | mmu-miR-183-3p | P05 | mmu-miR-3085-3p | P05 | Empty |  |  |  |
|  | P06 | mmu-miR-1839-3p | P06 | mmu-miR-1912-5p | P06 | Empty |  |  |  |
|  | P07 | mmu-miR-186-3p | P07 | mmu-miR-3104-5p | P07 | Empty |  |  |  |
|  | P08 | mmu-miR-1892 | P08 | mmu-miR-137-5p | P08 | Empty |  |  |  |
|  | P09 | mmu-miR-1895 | P09 | mmu-miR-3089-5p | P09 | Empty |  |  |  |
|  | P10 | mmu-miR-1896 | P10 | mmu-miR-3058-5p | P10 | Empty |  |  |  |
|  | P11 | mmu-miR-1897-3p | P11 | mmu-miR-3474 | P11 | Empty |  |  |  |
|  | P12 | mmu-miR-1897-5p | P12 | mmu-miR-125b-1-3p | P12 | Empty |  |  |  |
|  | P13 | **cel-miR-39-3p** | P13 | **cel-miR-39-3p** | P13 | **cel-miR-39-3p** |  |  |  |
|  | P14 | **cel-miR-39-3p** | P14 | **cel-miR-39-3p** | P14 | **cel-miR-39-3p** |  |  |  |
|  | P15 | **SNORD61** | P15 | **SNORD61** | P15 | **SNORD61** |  |  |  |
|  | P16 | **SNORD68** | P16 | **SNORD68** | P16 | **SNORD68** |  |  |  |
|  | P17 | **SNORD72** | P17 | **SNORD72** | P17 | **SNORD72** |  |  |  |
|  | P18 | **SNORD95** | P18 | **SNORD95** | P18 | **SNORD95** |  |  |  |
|  | P19 | **SNORD96A** | P19 | **SNORD96A** | P19 | **SNORD96A** |  |  |  |
|  | P20 | **RNU6-2** | P20 | **RNU6-2** | P20 | **RNU6-2** |  |  |  |
|  | P21 | **miRTC** | P21 | **miRTC** | P21 | **miRTC** |  |  |  |
|  | P22 | **miRTC** | P22 | **miRTC** | P22 | **miRTC** |  |  |  |
|  | P23 | **PPC** | P23 | **PPC** | P23 | **PPC** |  |  |  |
|  | P24 | **PPC** | P24 | **PPC** | P24 | **PPC** |  |  |  |
|  |  |  |  |  |  |  |  |  |  |
