# Supplementary material for: Identification of microRNAs as potential markers of ovarian toxicity
Source: J Appl Toxicol. 2018 Jan 29;38(5):744–52. doi: 10.1002/jat.3583 (PMC5901046; doi:10.1002/jat.3583)
Supplement: Supplementary file 5 — Supporting Information [file JAT-38-744-s005.docx]

**Gene Functional Classification Result**

**Control > Smoke**

**Gene Group 1 Enrichment Score: 4.8805332493470654**

**GENBANK_ACCESSION Gene Name**

NM_001081021 zinc finger protein 780B

NM_175175 pleckstrin homology domain containing, family F (with FYVE domain) member 2

NM_001076791 RIKEN cDNA 9630025I21 gene

NM_178607 ring finger protein 24

NM_009174 seven in absentia 2

NM_025962 methylmalonic aciduria cblC type, with homocystinuria

NM_019453 Mediterranean fever

NM_008904 peroxisome proliferative activated receptor, gamma, coactivator 1 alpha

NM_178728 N-acyl phosphatidylethanolamine phospholipase D

NM_027422 mesoderm induction early response 1, family member 2

NM_010127 POU domain, class 6, transcription factor 1

NM_001038610 dachshund 1 (Drosophila)

NM_138679 ash1 (absent, small, or homeotic)-like (Drosophila)

NM_011264 REV3-like, catalytic subunit of DNA polymerase zeta RAD54 like (S. cerevisiae)

NM_019747 zinc finger protein 113

NM_016746 cyclin C

NM_028538 RIKEN cDNA 1700049G17 gene

NM_010419 hairy and enhancer of split 5 (Drosophila)

NM_001033142 ring finger protein 166

NM_030713 zinc finger protein 202

NM_001080932 forkhead box K2

NM_011584 nuclear receptor subfamily 1, group D, member 2; predicted gene 5827

NM_138953 elongation factor RNA polymerase II 2

NM_001005358 zinc finger protein 97; cDNA sequence BC018101

NM_013692 Kruppel-like factor 10

NM_139141 zinc finger protein 192

NM_198601 tripartite motif-containing 52

NM_030256 B-cell CLL/lymphoma 9-like

NM_019706, NM_207623 ring finger protein 138

NM_011755 zinc finger protein 35

NM_010453 homeo box A5

NM_025895 mediator of RNA polymerase II transcription, subunit 28 homolog (yeast)

NM_172151 zinc finger, DHHC domain containing 8

NM_001025589, NM_011266 regulatory factor X-associated ankyrin-containing protein

NM_001048206, NM_011632 TNF receptor-associated factor 3

NM_021399 B-cell leukemia/lymphoma 11B

NM_031494 zinc finger protein 275

NM_172867 zinc finger protein 462

NM_013493 cellular nucleic acid binding protein

NM_198169 glucocorticoid modulatory element binding protein 2

NM_008736 neural retina leucine zipper gene

NM_144523 zinc finger protein 622

NM_177103 SUMO/sentrin specific peptidase 5

NM_080562 U box domain containing 5

NM_008686 nuclear factor, erythroid derived 2,-like 1

NM_177683 vestigial like 4 (Drosophila)

NM_008666 myelin transcription factor 1-like

NM_010053 distal-less homeobox 1

NM_178142 ligand dependent nuclear receptor corepressor-like

NM_029888 zinc finger protein 142

NM_172748 F-box and leucine-rich repeat protein 19

NM_172298 teashirt zinc finger family member 3

NM_001039214 mex3 homolog C (C. elegans)

NM_010797 similar to midline 1; midline 1

NM_175558 zinc finger protein 446

NM_028894 LON peptidase N-terminal domain and ring finger 3

NM_008236 hairy and enhancer of split 2 (Drosophila)

NM_194268 one cut domain, family member 2

NM_027485 mediator complex subunit 26

NM_001013756 grainyhead-like 3 (Drosophila)

NM_175557 zinc finger protein 112

NM_175341, NM_207515 muscleblind-like 2

NM_008612 menage a trois 1

NM_026647 zinc finger, DHHC domain containing 21

NM_177319 zinc finger, FYVE domain containing 27

NM_146040 cell division cycle associated 7 like

NM_001033813 zinc finger protein 872

NM_001081253 F-box protein 43

NM_144833 zinc finger protein 410

NM_133215 myotubularin related protein 4

NM_144873 ubiquitin-like, containing PHD and RING finger domains 2

NM_028372 metallo-beta-lactamase domain containing 2

NM_008780 paired box gene 1

NM_011546 zinc finger E-box binding homeobox 1

NM_001081409 PHD finger protein 20-like 1

NM_172303 PHD finger protein 17

NM_172717 checkpoint with forkhead and ring finger domains

NM_139226 one cut domain, family member 3

NM_029492 zinc finger, DHHC domain containing 20

NM_009385 NK2 homeobox 1

NM_011251, NM_029169 RNA binding motif protein 6

NM_008759 SEBOX homeobox

NM_011600 transducin-like enhancer of split 4, homolog of Drosophila E(spl)

NM_007880 AT rich interactive domain 3A (BRIGHT-like)

NM_019986 hyaluronic acid binding protein 4

NM_008541 MAD homolog 5 (Drosophila)

NM_153743, NM_007872 DNA methyltransferase 3A

NM_027642 PHD finger protein 6

NM_025389, NM_001038230 anaphase promoting complex subunit 11

NM_010791 mesenchyme homeobox 1

NM_197987 tripartite motif-containing 37

NM_172893 poly (ADP-ribose) polymerase family, member 12

NM_011355 SFFV proviral integration 1

NM_080433 Fez family zinc finger 2

NM_001033469 forkhead box R1

NM_009032 RNA binding motif protein 4

NM_007562 basonuclin 1

NM_178115 RIKEN cDNA 2700050L05 gene

NM_011935 estrogen-related receptor gamma

NM_020493 serum response factor

NM_001025560, NM_011445 SRY-box containing gene 6

NM_133919 AF4/FMR2 family, member 1

NM_145596 similar to Transcriptional repressor p66 alpha (GATA zinc finger domain-containing protein 2A); GATA zinc finger domain containing 2A

NM_199196 suppressor of zeste 12 homolog (Drosophila)

NM_033327 zinc finger protein 423; similar to mKIAA0760 protein

NM_011753 zinc finger protein 26

NM_176850 bromodomain PHD finger transcription factor

NM_133817 zinc finger protein 451

NM_020267 tripartite motif-containing 44

NM_198417 RIKEN cDNA C030039L03 gene

NM_146175 zinc finger protein 282

NM_172539 similar to Astacin-like metalloendopeptidase precursor (Oocyte astacin) (Ovastacin); astacin-like metalloendopeptidase (M12 family)

NM_008961 phosphotriesterase related

NM_001013392 ras responsive element binding protein 1

NM_177790 zinc finger protein 385C

NM_026406 similar to Rabring 7; ring finger protein 115

NM_001001182 bromodomain adjacent to zinc finger domain, 2B

NM_001025577 similar to c-Maf long form; avian musculoaponeurotic fibrosarcoma (v-maf) AS42 oncogene homolog

NM_028716 PHD finger protein 19

NM_001004143 ubiquitin specific peptidase 22

NM_001009935 thioredoxin interacting protein

NM_178609 E2F transcription factor 7

NM_028190 Luc7 homolog (S. cerevisiae)-like

NM_028298 zinc finger protein 655

NM_007678 CCAAT/enhancer binding protein (C/EBP), alpha

NM_133784 WW domain containing transcription regulator 1

NM_133957 nuclear factor of activated T-cells 5

NM_133218 zinc finger protein 704

NM_001037940 DnaJ (Hsp40) homolog, subfamily B, member 6; predicted gene 5917; predicted gene 15785; predicted gene 4852; predicted gene 2541

NM_001045486 similar to Cys2/His2 zinc finger protein (rKr1); zinc finger protein 180

NM_019743 RING1 and YY1 binding protein

NM_025883, NM_001033573 predicted gene 5509; zinc finger, DHHC domain containing 6

NM_027504 PR domain containing 16

NM_001039048 similar to tripartite motif-containing 63; tripartite motif-containing 63

NM_173780 Kruppel-like factor 8

NM_022722 dihydropyrimidinase

NM_145511, NM_001077237 similar to odorant response abnormal 4; cDNA sequence BC003331

NM_007913 early growth response 1

NM_007496 zinc finger homeobox 3

NM_011308 nuclear receptor co-repressor 1

NM_008676 neighbor of Brca1 gene 1

NM_001012330 zinc finger protein 238

NM_144937 ubiquitin specific peptidase 3

NM_009397 tumor necrosis factor, alpha-induced protein 3

NM_010155 Ets2 repressor factor

NM_009517 zinc finger matrin type 3

NM_133206 zinc and ring finger 1

NM_175494 zinc finger protein 367

NM_144783 similar to Wilms tumor homolog; Wilms tumor 1 homolog

NM_026949 CCR4-NOT transcription complex, subunit 8

NM_177239 similar to mKIAA1915 protein; myb-like, SWIRM and MPN domains 1

NM_207222 LIM domain only 3

NM_009239 trans-acting transcription factor 4

NM_021878 jumonji, AT rich interactive domain 2

NM_019812 sirtuin 1 (silent mating type information regulation 2, homolog) 1 (S. cerevisiae)

NM_138944 POU domain, class 4, transcription factor 2

NM_173769 zinc finger protein 641

NM_001008543, NM_010847 Max interacting protein 1

NM_025729 mitogen-activated protein kinase kinase kinase 7 interacting protein 3

NM_023465 catenin beta interacting protein 1

NM_008186 general transcription factor II H, polypeptide 1; similar to 62 kDa subunit of TFIIH

NM_199449 zinc fingers and homeoboxes 2

NM_024495 carbonic anhydrase 13

NM_001039081, NM_027696 mesoderm induction early response 1 homolog (Xenopus laevis

NM_009575 zinc finger protein of the cerebellum 3

NM_009566 zinc finger protein 92

NM_009031 retinoblastoma binding protein 7; predicted gene 6382

NM_009939 COP9 (constitutive photomorphogenic) homolog, subunit 2 (Arabidopsis thaliana)

NM_030564 ring finger protein 34

NM_007935, NM_027497 enhancer of polycomb homolog 1 (Drosophila)

NM_012037 vesicle amine transport protein 1 homolog (T californica)

NM_010264 nuclear receptor subfamily 6, group A, member 1

NM_009822 runt-related transcription factor 1; translocated to, 1 (cyclin D-related)

NM_178779 ring finger protein 152

NM_008652 myeloblastosis oncogene-like 2

NM_009234 SRY-box containing gene 11

NM_011762 zinc finger protein 59; predicted gene 7452

NM_172860 core-binding factor, runt domain, alpha subunit 2, translocated to, 2 (human)

NM_145487 proline-rich polypeptide 3

NM_013508 ELK3, member of ETS oncogene family

NM_198035 zinc finger and BTB domain containing 39

NM_013720 MAX gene associated

NM_008263 homeo box A10

NM_001038642, NM_011808 E26 avian leukemia oncogene 1, 5' domain

NM_172606 membrane-associated ring finger (C3HC4) 6

NM_012051 ets variant gene 3

NM_178375 zinc finger, SWIM domain containing 3

NM_027425 RUN and FYVE domain-containing 2

NM_001081269 Wolf-Hirschhorn syndrome candidate 1-like 1 (human)

NM_175647 doublesex and mab-3 related transcription factor like family A1

NM_146028 SH3 and cysteine rich domain 2

NM_008539 MAD homolog 1 (Drosophila)

NM_033270 E2F transcription factor 6

NM_027658 similar to hexamthylene bis-acetamide inducible 2; hexamthylene bis-acetamide inducible 2

NM_153802 zinc finger protein 128

NM_028487 GC-rich promoter binding protein 1

NM_021346 zinc finger protein 318

NM_027973 myeloid leukemia factor 1 interacting protein

NM_026856 zinc finger protein 644

NM_010938 nuclear respiratory factor 1

NM_153780 RIKEN cDNA 2610044O15 gene

NM_007626 chromobox homolog 5 (Drosophila HP1a)

NM_025844 cysteine and histidine-rich domain (CHORD)-containing, zinc-binding protein 1

NM_001003909 ankyrin repeat and IBR domain containing 1

NM_025483, NM_001003971 SUMO1/sentrin specific peptidase 7

NM_028774 ring finger protein (C3H2C3 type) 6

NM_001039090, NM_011386 SKI-like

NM_028039 establishment of cohesion 1 homolog 2 (S. cerevisiae)

NM_016889 insulinoma-associated 1

NM_025283 MOB1, Mps One Binder kinase activator-like 3 (yeast)

NM_177884 expressed sequence AW146020

NM_177342 TAF5 RNA polymerase II, TATA box binding protein (TBP)-associated factor

NM_175311 zinc finger protein 513

NM_013594 methyl-CpG binding domain protein 1

NM_010919 NK2 transcription factor related, locus 2 (Drosophila)

NM_145624 adenosine deaminase, tRNA-specific 1

NM_172269 vacuolar protein sorting 18 (yeast)

NM_016660, NM_001025427 high mobility group AT-hook I, related sequence 1; high mobility group AT-hook 1

NM_008037 similar to fos-like antigen 2; fos-like antigen 2

NM_013745 nuclear fragile X mental retardation protein interacting protein 1

NM_028141 zinc finger protein 661

NM_199422 S100 calcium binding protein A7A

**Gene Group 2 Enrichment Score: 3.603477064701271**

**GENBANK_ACCESSION Gene Name**

NM_027297 PRP4 pre-mRNA processing factor 4 homolog (yeast)

NM_025897 ribosomal RNA processing 8, methyltransferase, homolog (yeast)

NM_011358 splicing factor, arginine/serine-rich 2 (SC-35)

NM_026631 NHP2 ribonucleoprotein homolog (yeast)

NM_021714 WW domain binding protein 11

NM_001078167, NM_173374 splicing factor, arginine/serine-rich 1 (ASF/SF2); similar to splicing factor, arginine/serine-rich 1 (splicing factor 2, alternate splicing factor)

NM_001081956 splicing factor, arginine/serine-rich 17b

NM_026538 DEAD (Asp-Glu-Ala-Asp) box polypeptide 56

NM_025822 arginine/serine-rich coiled-coil 1

**Gene Group 3 Enrichment Score: 3.591105855666617**

**GENBANK_ACCESSION Gene Name**

NM_024436 RAB22A, member RAS oncogene family

NM_017380 septin 9

NM_026697 RAB14, member RAS oncogene family

NM_175092 ras homolog gene family, member f

NM_172413 similar to RAP2C, member of RAS oncogene family; RAP2C, member of RAS oncogene family

NM_029576 RAB1B, member RAS oncogene family

NM_175562 RAB39, member RAS oncogene family

NM_032544 GTP binding protein 3

NM_053173 similar to Kifc1 protein; kinesin family member C1; predicted gene 4137

NM_053075 Ras homolog enriched in brain; similar to RAS-homolog enriched in brain

NM_173781 RAB6B, member RAS oncogene family

NM_010306 guanine nucleotide binding protein (G protein), alpha inhibiting 3

NM_011889 septin 3

NM_172601 RAB2B, member RAS oncogene family

NM_145491 ras homolog gene family, member Q

NM_001024911 septin 10

NM_178119, NM_001037136 ArfGAP with GTPase domain, ankyrin repeat and PH domain 1

NM_016676 RAB10, member RAS oncogene family

NM_024457 RAS related protein 1b; similar to GTP-binding protein (smg p21B)

NM_007476 predicted gene 5823; ADP-ribosylation factor 1; predicted gene 8230

NM_022327 v-ral simian leukemia viral oncogene homolog B (ras related)

NM_026817 RAB, member of RAS oncogene family-like 2A

NM_011655 tubulin, beta 5

NM_176971 RAB9B, member RAS oncogene family

NM_024216 GPN-loop GTPase 3

NM_019942 septin 6

NM_145541 predicted gene 9392; similar to Raichu404X; RAS-related protein-1a

NM_177411 RAB5B, member RAS oncogene family; predicted gene 13991; similar to RAB5B, member RAS oncogene family

NM_007478 ADP-ribosylation factor 3

NM_025887 RAB5A, member RAS oncogene family; similar to small GTP-binding protein rab5

NM_173363 similar to Eukaryotic translation initiation factor 5; eukaryotic translation initiation factor 5

NM_028006 epsilon-tubulin 1

NM_008997 RAB11B, member RAS oncogene family

NM_026011 ADP-ribosylation factor-like 8B

NM_001039394, NM_133717 RAB43, member RAS oncogene family

NM_026130 signal recognition particle receptor ('docking protein')

**Gene Group 4 Enrichment Score: 3.1845338965577152**

**GENBANK_ACCESSION Gene Name**

NM_009032 RNA binding motif protein 4

NM_001081956 splicing factor, arginine/serine-rich 17b

NM_019550 polypyrimidine tract binding protein 2

NM_025690 SAFB-like, transcription modulator

NM_178164, NM_144904 ROD1 regulator of differentiation 1 (S. pombe)

NM_001003899, NM_001008546, NM_145556, NM_001008545 predicted gene 13886; TAR DNA binding protein

NM_053104 RNA binding motif protein 9

NM_019733 RNA binding protein gene with multiple splicing

NM_146130 predicted gene 7498; hypothetical protein LOC635773; predicted gene 9242; predicted gene 6793; predicted gene 14730; predicted gene 7551; predicted gene 14398; predicted gene 6528; predicted gene 6641; hypothetical protein LOC630507; predicted gene 5550; predicted gene 11847; predicted gene 6506; predicted gene 6153; predicted gene 5469; predicted gene 8991; similar to heterogeneous nuclear ribonucleoprotein A3; predicted gene 6758; predicted gene, OTTMUSG00000009698; heterogeneous nuclear ribonucleoprotein A3; predicted gene 5896

NM_026434 RNA binding motif protein 18

NM_010880 nucleolin

NM_001078167, NM_173374 splicing factor, arginine/serine-rich 1 (ASF/SF2); similar to splicing factor, arginine/serine-rich 1 (splicing factor 2, alternate splicing factor)

NM_170590, NM_170588 copine I

NM_019711, NM_001039080 RNA binding motif, single stranded interacting protein 2

NM_016856 cleavage and polyadenylation specific factor 2

NM_010160 CUG triplet repeat, RNA binding protein 2

NM_198102 transformer 2 alpha homolog (Drosophila)

NM_146083 splicing factor, arginine/serine-rich 7

NM_172302 cleavage and polyadenylation specific factor 7

NM_011358 splicing factor, arginine/serine-rich 2 (SC-35)

**Gene Group 5 Enrichment Score: 2.9307791246591557**

**GENBANK_ACCESSION Gene Name**

NM_019653 WD repeat and SOCS box-containing 1

NM_172871 kelch-like 9 (Drosophila)

NM_022022 ubiquitination factor E4B, UFD2 homolog (S. cerevisiae)

NM_146193 similar to BTB (POZ) domain containing 1 (predicted); BTB (POZ) domain containing 1; predicted gene 14121

NM_198636 acyl-CoA synthetase short-chain family member 3

NM_022995 prostate transmembrane protein, androgen induced 1; similar to Nedd4 WW binding protein 4

NM_001081253 F-box protein 43

NM_028705 hect domain and RLD 3

NM_027604 ubiquitin specific peptidase 15

NM_001008785 kelch repeat and BTB (POZ) domain containing 8

NM_001033634 zyg-ll homolog B (C. elegans)

NM_172518 F-box protein 42

NM_172606 membrane-associated ring finger (C3HC4) 6

NM_145420 ubiquitin-conjugating enzyme E2D 1, UBC4/5 homolog (yeast)

NM_207238 F-box protein 27

NM_009481 ubiquitin specific peptidase 9, X chromosome

NM_029749 ubiquitin specific peptidase 42

NM_145958 kelch repeat and BTB (POZ) domain containing 2

NM_029402 cullin 2

NM_019929 SMT3 suppressor of mif two 3 homolog 3 (yeast)

NM_001038589, NM_021522 ubiquitin specific peptidase 14

NM_025389, NM_001038230 anaphase promoting complex subunit 11

NM_029773 speckle-type POZ protein-like

NM_019706, NM_207623 ring finger protein 138

NM_025985 ubiquitin-conjugating enzyme E2G 1 (UBC7 homolog, C. elegans)

NM_173754 ubiquitin specific peptidase 43

NM_080562 U box domain containing 5

NM_177103 SUMO/sentrin specific peptidase 5

NM_175029 hypothetical protein LOC100044217; autophagy-related 4C (yeast)

NM_025483, NM_001003971 SUMO1/sentrin specific peptidase 7

NM_025356 ubiquitin-conjugating enzyme E2D 3 (UBC4/5 homolog, yeast); similar to UBE2D3; predicted gene 4596; predicted gene 15361

NM_009397 tumor necrosis factor, alpha-induced protein 3

NM_153128 kelch-like 12 (Drosophila)

NM_133907 ubiquitin protein ligase E3C

NM_030197 RIKEN cDNA 2700078E11 gene

NM_009456 predicted gene 7423; predicted gene 5858; predicted gene 6562; similar to ubiquitin-conjugating enzyme UbcH7; predicted gene 3076; ubiquitin-conjugating enzyme E2L 3; predicted gene 15267; predicted gene 9057; predicted gene 10705; predicted gene 10145

NM_148949 TD and POZ domain containing 1; similar to TD and POZ domain-containing protein 1 (MAPP family protein 2); predicted gene 9117; predicted gene 9125

NM_009174 seven in absentia 2

NM_144937 ubiquitin specific peptidase 3

NM_173443 valosin containing protein (p97)/p47 complex interacting protein 1

NM_001004143 ubiquitin specific peptidase 22

**Gene Group 6 Enrichment Score: 2.6606375641520668**

**GENBANK_ACCESSION Gene Name**

NM_001005475 IQ motif and Sec7 domain 2

NM_194334 TBC1 domain family, member 2B

NM_011180 cytohesin 1

NM_177698 pleckstrin and Sec7 domain containing 3

NM_028195 cytohesin 4

NM_001033258 DNA segment, Chr 10, Brigham & Women's Genetics 1379 expressed

**Gene Group 7 Enrichment Score: 2.5646726451236264**

**GENBANK_ACCESSION Gene Name**

NM_028127 predicted gene 5780; FERM domain containing 6

NM_009041 radixin

NM_008976 protein tyrosine phosphatase, non-receptor type 14

NM_011204 protein tyrosine phosphatase, non-receptor type 13

NM_145148 FERM domain containing 4B

NM_001006664, NM_013510, NM_001003815 erythrocyte protein band 4.1-like 1

**Gene Group 8 Enrichment Score: 2.414393135135399**

**GENBANK_ACCESSION Gene Name**

NM_023871 predicted gene, EG625349; predicted gene 5789; predicted gene 7085; predicted gene 5708; predicted gene 6847; SET translocation; cDNA sequence BC085271; predicted gene 7239; similar to protein phosphatase 2A inhibitor-2 I-2PP2A; predicted gene 9531

NM_021432 nucleosome assembly protein 1-like 5

NM_178194 histone cluster 1, H2bg; histone cluster 1, H2be; histone cluster 2, H2bb; histone cluster 1, H2bc

NM_009433 testis-specific protein, Y-encoded-like 1

**Gene Group 9 Enrichment Score: 2.407525717864325**

**GENBANK_ACCESSION Gene Name**

NM_011200 similar to tyrosine phosphatase; predicted gene 8783; predicted gene 15801; predicted gene 5951; protein tyrosine phosphatase 4a1; similar to killer cell lectin-like receptor subfamily A member 2

NM_008974 predicted gene 13422; protein tyrosine phosphatase 4a2

NM_011203 protein tyrosine phosphatase, non-receptor type 12

NM_008976 protein tyrosine phosphatase, non-receptor type 14

NM_019637 serine/threonine/tyrosine interaction protein; predicted gene 14698

NM_011204 protein tyrosine phosphatase, non-receptor type 13

NM_177081 protein tyrosine phosphatase, non-receptor type 7

**Gene Group 10 Enrichment Score: 2.265789991734906**

**GENBANK_ACCESSION Gene Name**

NM_172717 checkpoint with forkhead and ring finger domains

NM_019499 similar to spindle assembly checkpoint protein; MAD2 mitotic arrest deficient-like 1 (yeast)

NM_009481 ubiquitin specific peptidase 9, X chromosome

NM_021465, NM_001077712 stromal antigen 2

NM_007635 cyclin G2

NM_053173 similar to Kifc1 protein; kinesin family member C1; predicted gene 4137

NM_010441 predicted gene 7996; high mobility group AT-hook 2

NM_026904 anaphase promoting complex subunit 10

NM_025389, NM_001038230 anaphase promoting complex subunit 11

NM_009282 stromal antigen 1

**Gene Group 11 Enrichment Score: 2.2069110662031437**

**GENBANK_ACCESSION Gene Name**

NM_028959 centrosomal protein 72

NM_019785 ARP10 actin-related protein 10 homolog (S. cerevisiae)

NM_024191 ADP-ribosylation factor-like 2 binding protein

NM_001039179, NM_029791, NM_001039180 bicaudal D homolog 2 (Drosophila)

**Gene Group 12 Enrichment Score: 2.1903767023998775**

**GENBANK_ACCESSION Gene Name**

NM_146124 Rho GTPase activating protein 1; predicted gene 8514

NM_001005508 Rho GTPase activating protein 30

NM_019834 G protein-coupled receptor kinase-interactor 2

NM_019994 GTPase activating RANGAP domain-like 1

NM_026254 TBC1 domain family, member 23

NM_025706 TBC1 domain family, member 15

NM_194334 TBC1 domain family, member 2B

NM_173186 TBC1 domain family, member 24

NM_028162 TBC1 domain family, member 5

NM_007486 Rho, GDP dissociation inhibitor (GDI) beta

**Gene Group 13 Enrichment Score: 2.139491650143279**

**GENBANK_ACCESSION Gene Name**

NM_153774 importin 9

NM_023146 RAN binding protein 17

NM_010655 similar to ribosomal protein L38; predicted gene 13020; ribosomal protein L38; predicted gene 4991; karyopherin (importin) alpha 2; predicted gene 9028; predicted gene 8129; predicted gene 7123; predicted gene 5832; predicted gene 10184; predicted gene 7379; predicted gene 10259

NM_178716, NM_001048267 predicted gene 6493; transportin 1

NM_008379 karyopherin (importin) beta 1

**Gene Group 14 Enrichment Score: 1.804506461312278**

**GENBANK_ACCESSION Gene Name**

NM_153587 ribosomal protein S6 kinase, polypeptide 5

NM_018737 cytidine 5'-triphosphate synthase 2

NM_011103 protein kinase C, delta

NM_016866 serine/threonine kinase 39, STE20/SPS1 homolog (yeast)

NM_198636 acyl-CoA synthetase short-chain family member 3

NM_144551 tribbles homolog 2 (Drosophila)

NM_173185 casein kinase 1, gamma 1

NM_011785 thymoma viral proto-oncogene 3

NM_183162 cDNA sequence BC006779

NM_001004363 NUAK family, SNF1-like kinase, 1

NM_177326 p21 protein (Cdc42/Rac)-activated kinase 2

NM_199056 inositol 1,3,4,5,6-pentakisphosphate 2-kinase

NM_007395 activin A receptor, type 1B

NM_013830 PRP4 pre-mRNA processing factor 4 homolog B (yeast)

NM_019777 inhibitor of kappaB kinase epsilon

NM_009793 calcium/calmodulin-dependent protein kinase IV

NM_019635 serine/threonine kinase 3 (Ste20, yeast homolog)

NM_172134 pyridoxal (pyridoxine, vitamin B6) kinase

NM_008445 kinesin family member 3C

NM_011163 eukaryotic translation initiation factor 2-alpha kinase 2

NM_019978 doublecortin-like kinase 1

NM_146243 ARP2 actin-related protein 2 homolog (yeast); predicted gene 6828

NM_011361 serum/glucocorticoid regulated kinase 1

NM_007434 similar to RAC-beta serine/threonine-protein kinase (RAC-PK-beta) (Protein kinase Akt-2) (Protein kinase B, beta) (PKB beta); thymoma viral proto-oncogene 2; similar to serine/threonine kinase

NM_133823 methylmalonic aciduria (cobalamin deficiency) type A

NM_008809 platelet derived growth factor receptor, beta polypeptide

NM_019827 glycogen synthase kinase 3 beta

NM_133741 SNF related kinase; hypothetical protein LOC100044493; predicted gene 3193

NM_172555 poly(A) polymerase gamma

NM_007936 Eph receptor A4

NM_028126 STE20-related kinase adaptor alpha

NM_001038635, NM_183262 serine/threonine kinase 35

NM_009266 selenophosphate synthetase 2

NM_010717 LIM-domain containing, protein kinase

NM_007982 PTK2 protein tyrosine kinase 2

NM_001025074 neurotrophic tyrosine kinase, receptor, type 2

NM_009516 WEE 1 homolog 1 (S. pombe)

NM_009703 v-raf murine sarcoma 3611 viral oncogene homolog

NM_013881 Unc-51 like kinase 2 (C. elegans)

NM_021299 adenylate kinase 3

NM_147201 nuclear receptor binding protein 1

NM_177390 myosin ID

NM_198703 WNK lysine deficient protein kinase 1

NM_029653 death associated protein kinase 1

NM_007463 SPEG complex locus

NM_010583 IL2-inducible T-cell kinase

NM_001013367 protein kinase, AMP-activated, alpha 1 catalytic subunit

NM_175460 nicotinamide nucleotide adenylyltransferase 2

NM_025985 ubiquitin-conjugating enzyme E2G 1 (UBC7 homolog, C. elegans)

NM_028291 PAN3 polyA specific ribonuclease subunit homolog (S. cerevisiae)

NM_025949 ribosomal protein S6 kinase polypeptide 6

NM_001033369 activin A receptor, type IC

NM_025356 ubiquitin-conjugating enzyme E2D 3 (UBC4/5 homolog, yeast); similar to UBE2D3; predicted gene 4596; predicted gene 15361

NM_029031 sedoheptulokinase

NM_027184 inositol polyphosphate multikinase

NM_009068 receptor (TNFRSF)-interacting serine-threonine kinase 1

NM_172656 STE20-related kinase adaptor beta

NM_009456 predicted gene 7423; predicted gene 5858; predicted gene 6562; similar to ubiquitin-conjugating enzyme UbcH7; predicted gene 3076; ubiquitin-conjugating enzyme E2L 3; predicted gene 15267; predicted gene 9057; predicted gene 10705; predicted gene 10145

NM_021716 fidgetin

NM_011951 mitogen-activated protein kinase 14

NM_011112 poly (A) polymerase alpha

NM_198004, NM_001048060 RIKEN cDNA 5133401N09 gene

**Gene Group 15 Enrichment Score: 1.72220855681662**

**GENBANK_ACCESSION Gene Name**

NM_001033332 transmembrane and tetratricopeptide repeat containing 3

NM_053217 RIKEN cDNA 2010002M12 gene

NM_025978 tetratricopeptide repeat domain 14

NM_001033149 tetratricopeptide repeat domain 9

**Gene Group 16 Enrichment Score: 1.668522890817093**

**GENBANK_ACCESSION Gene Name**

NM_178373 cell death-inducing DFFA-like effector c

NM_021347 gasdermin A

NM_009811 caspase 6

NM_009809 caspase 14

NM_021897 transformation related protein 53 inducible nuclear protein 1

NM_009805 CASP8 and FADD-like apoptosis regulator pseudogene; CASP8 and FADD-like apoptosis regulator

NM_015733 caspase 9

**Gene Group 17 Enrichment Score: 1.5605079030670586**

**GENBANK_ACCESSION Gene Name**

NM_009408 topoisomerase (DNA) I

NM_144958 eukaryotic translation initiation factor 4A1

NM_026538 DEAD (Asp-Glu-Ala-Asp) box polypeptide 56

NM_028041 DEAD (Asp-Glu-Ala-Asp) box polypeptide 54

NM_029337 E1A binding protein p400

NM_021716 fidgetin

**Gene Group 18 Enrichment Score: 1.4131770635626963**

**GENBANK_ACCESSION Gene Name**

NM_011600 transducin-like enhancer of split 4, homolog of Drosophila E(spl)

NM_027946 WD repeat domain 68

NM_019653 WD repeat and SOCS box-containing 1

NM_145125 bromodomain and WD repeat domain containing 1

NM_027297 PRP4 pre-mRNA processing factor 4 homolog (yeast)

NM_010063 dynein cytoplasmic 1 intermediate chain 1

NM_145374 missing oocyte, meiosis regulator, homolog (Drosophila)

NM_175484 coronin, actin binding protein, 2B

NM_030234 WD repeat domain 76

NM_009030 retinoblastoma binding protein 4

NM_177342 TAF5 RNA polymerase II, TATA box binding protein (TBP)-associated factor

NM_011715 WD repeat domain 1

NM_001081216 pleckstrin homology domain interacting protein

NM_199466 echinoderm microtubule associated protein like 4

**Gene Group 19 Enrichment Score: 1.1182122827724998**

**GENBANK_ACCESSION Gene Name**

NM_172539 similar to Astacin-like metalloendopeptidase precursor (Oocyte astacin) (Ovastacin); astacin-like metalloendopeptidase (M12 family)

NM_021362 pregnancy-associated plasma protein A

NM_009613 a disintegrin and metallopeptidase domain 11

NM_011780 a disintegrin and metallopeptidase domain 23; similar to ADAM23

NM_008605 matrix metallopeptidase 12

NM_175506 a disintegrin-like and metallopeptidase (reprolysin type) with thrombospondin type 1 motif, 19

NM_178396 carbonic anyhydrase 12

**Gene Group 20 Enrichment Score: 1.0683583450430134**

**GENBANK_ACCESSION Gene Name**

NM_025850 fibronectin type 3 and ankyrin repeat domains 1

NM_029885, NM_172512 GA repeat binding protein, beta 2

NM_181413 ankyrin repeat and SAM domain containing 1

NM_133971 ankyrin repeat domain 10

NM_207669 GA repeat binding protein, beta 1

NM_025980 Notch-regulated ankyrin repeat protein

**Gene Group 21 Enrichment Score: 0.8992532673675001**

**GENBANK_ACCESSION Gene Name**

NM_019758 mitochondrial carrier homolog 2 (C. elegans); predicted gene, 100039384; predicted gene, 100039506

NM_016804 metaxin 2

NM_172609 predicted gene 12906; predicted gene 7250; translocase of outer mitochondrial membrane 22 homolog (yeast)

NM_181325 solute carrier family 25 (mitochondrial carrier ornithine transporter), member 15

NM_026950 OCIA domain containing 2

NM_025292 RIKEN cDNA 1810020G14 gene; synaptojanin 2 binding protein; predicted gene 4116

**Gene Group 22 Enrichment Score: 0.7894019177499446**

**GENBANK_ACCESSION Gene Name**

NM_138652 ATPase, H+/K+ transporting, nongastric, alpha polypeptide

NM_001037863, NM_001001798 ATPase, class VI, type 11C

NM_018757 similar to Nme6 protein; non-metastatic cells 6, protein expressed in (nucleoside-diphosphate kinase)

NM_026482 ATPase, Ca++ transporting, plasma membrane 1

**Gene Group 23 Enrichment Score: 0.6285419161140905**

**GENBANK_ACCESSION Gene Name**

NM_028643 EF hand domain family A1

NM_013877 similar to CaBP5; calcium binding protein 5

NM_199422 S100 calcium binding protein A7A

NM_178256 RALBP1 associated Eps domain containing protein 2

NM_010471 hippocalcin

NM_009037 reticulocalbin 1

**Gene Group 24 Enrichment Score: 0.5721250177735306**

**GENBANK_ACCESSION Gene Name**

NM_013822 jagged 1

NM_172463 sushi, nidogen and EGF-like domains 1

NM_007789 neurocan; similar to Neurocan

NM_022723 signal peptide, CUB domain, EGF-like 1

NM_008695 nidogen 2

**Gene Group 25 Enrichment Score: 0.34602294038817666**

**GENBANK_ACCESSION Gene Name**

NM_145517 ORM1-like 1 (S. cerevisiae)

NM_178607 ring finger protein 24

NM_198702 latrophilin 3

NM_025965 signal sequence receptor, alpha; similar to signal sequence receptor, alpha

NM_145925 pituitary tumor-transforming 1 interacting protein

NM_021324 tweety homolog 1 (Drosophila)

NM_026331 solute carrier family 25, member 37

NM_001033633 solute carrier family 2 (facilitated glucose transporter), member 13

NM_207633 similar to Yip1 domain family, member 6; Yip1 domain family, member 6

NM_172616 RIKEN cDNA C330027C09 gene

NM_010279 glial cell line derived neurotrophic factor family receptor alpha 1

NM_153506 C-type lectin domain family 2, member e

NM_177664 DNA segment, Chr 3, Brigham & Women's Genetics 0562 expressed

NM_145426 microfibrillar-associated protein 3

NM_001033393 transmembrane protein 104

NM_011924 arginine vasopressin receptor 1B

NM_008177 gastrin releasing peptide receptor

NM_019510 transient receptor potential cation channel, subfamily C, member 3

NM_205810 MAS-related GPR, member B1

NM_021464 protein tyrosine phosphatase, receptor type, T

NM_009403 tumor necrosis factor (ligand) superfamily, member 8

NM_026239 transmembrane protein 35

NM_001001738 inositol 1,4,5-triphosphate receptor interacting protein

NM_175519 potassium channel tetramerisation domain containing 8

NM_020028 lysophosphatidic acid receptor 2

NM_172435 purinergic receptor P2Y, G-protein coupled 10

NM_033149 UDP-Gal:betaGlcNAc beta 1,3-galactosyltransferase, polypeptide 5

NM_009183 ST8 alpha-N-acetyl-neuraminide alpha-2,8-sialyltransferase 4

NM_027379, NM_026143 fatty acyl CoA reductase 1

NM_172620 vacuolar protein sorting 52 (yeast)

NM_008421 potassium voltage gated channel, Shaw-related subfamily, member 1

NM_025708 transmembrane protein 186

NM_029394 sorting nexing 24

NM_145502 ER lipid raft associated 1

NM_172151 zinc finger, DHHC domain containing 8

NM_001033759 transmembrane protein 2

NM_146698 olfactory receptor 1443

NM_022995 prostate transmembrane protein, androgen induced 1; similar to Nedd4 WW binding protein 4

NM_134060 solute carrier family 35, member B3

NM_028793 acyl-Coenzyme A binding domain containing 5

NM_026685 transmembrane protein 174

NM_145838 ST8 alpha-N-acetyl-neuraminide alpha-2,8-sialyltransferase 6

NM_181415 attractin like 1

NM_001004173 sphingosine-1-phosphate phosphotase 2

NM_172383 transmembrane protein 125

NM_027052 solute carrier family 38, member 4

NM_015775 transmembrane protease, serine 2

NM_173212, NM_027454 cholinergic receptor, nicotinic, beta polypeptide 3

NM_010585 inositol 1,4,5-triphosphate receptor 1

NM_028343 transmembrane protein 135

NM_016898 CD164 antigen

NM_177366 similar to G protein-coupled receptor 157; G protein-coupled receptor 157

NM_001033332 transmembrane and tetratricopeptide repeat containing 3

NM_009472 unc-5 homolog C (C. elegans)

NM_016663 synaptotagmin III

NM_013635, NM_198710 synaptophysin-like protein

NM_172621 chloride intracellular channel 5

NM_001003917 autophagy-related 9A (yeast)

NM_178714 leucine rich repeat and fibronectin type III domain containing 5

NM_001081186 RIKEN cDNA D630042F21 gene

NM_019413 roundabout homolog 1 (Drosophila)

NM_007911 ephrin B3

NM_025813 major facilitator superfamily domain containing 1

NM_001013411 Na+/K+ transporting ATPase interacting 2

NM_023587 predicted gene 7908; protein tyrosine phosphatase-like (proline instead of catalytic arginine), member b

NM_199021 dipeptidylpeptidase 10

NM_001004151 receptor transporter protein 1

NM_009730 attractin

NM_133733 RIKEN cDNA 9030425E11 gene

NM_009223 stannin

NM_145853 two pore channel 1

NM_020611 steroid 5 alpha-reductase 3; hypothetical protein LOC100044230

NM_026673 similar to Novel transmembrane domain containing protein; apolipoprotein O

NM_001012517 fucosyltransferase 10

NM_007538 opsin 1 (cone pigments), short-wave-sensitive (color blindness, tritan)

NM_138602 PRA1 domain family 2; predicted gene 4168

NM_026647 zinc finger, DHHC domain containing 21

NM_025889, NM_001078649 transmembrane protein 134

NM_178592 HLA-B associated transcript 5

NM_172479 solute carrier family 38, member 5

NM_177319 zinc finger, FYVE domain containing 27

NM_133718 transmembrane protein 30A

NM_175316 solute carrier organic anion transporter family, member 2b1

NM_001033468 G protein-coupled receptor 114

NM_015799 transferrin receptor 2

NM_011394 hypothetical protein LOC100045882; solute carrier family 20, member 2

NM_133352 transmembrane 9 superfamily member 3

NM_021530 solute carrier family 4 (anion exchanger), member 8

NM_010763 mannosidase, alpha, class 1A, member 2

NM_026343 syntaxin 17

NM_023396 reprimo, TP53 dependent G2 arrest mediator candidate

NM_175130 transient receptor potential cation channel, subfamily M, member 4

NM_029492 zinc finger, DHHC domain containing 20

NM_172051 transmembrane and coiled coil domains 3

NM_033564 Mpv17 transgene, kidney disease mutant-like

NM_001025581 potassium voltage gated channel, Shaw-related subfamily, member 2

NM_178886 low density lipoprotein receptor class A domain containing 3

NM_172608 transmembrane protein 184b

NM_153581 glycoprotein m6a

NM_183160 RIKEN cDNA E030010A14 gene

NM_145129 cholinergic receptor, nicotinic, alpha polypeptide 3

NM_009924 cannabinoid receptor 2 (macrophage)

NM_144821 expressed sequence AI317395

NM_172706 RIKEN cDNA 9330182L06 gene

NM_001001985 N-acetyltransferase 8-like

NM_173048 golgi associated, gamma adaptin ear containing, ARF binding protein 3

NM_027533 tetraspanin 2

NM_144798 solute carrier family 30 (zinc transporter), member 6

NM_028166 RIKEN cDNA 1600014C10 gene

NM_011512 surfeit gene 4

NM_023680 tumor necrosis factor receptor superfamily, member 22

NM_172145 family with sequence similarity 176, member B

NM_183427 glycine receptor, alpha 2 subunit

NM_029098 limb region 1 like

NM_019697 potassium voltage-gated channel, Shal-related family, member 2

NM_016869 corin

NM_170593 dispatched homolog 2 (Drosophila)

NM_153529 neuritin 1

NM_178005 leucine rich repeat transmembrane neuronal 2

NM_028995 NIPA-like domain containing 3; similar to NIPA-like domain containing 3

NM_008313 5 hydroxytryptamine (serotonin) receptor 4

NM_008124 gap junction protein, beta 1

NM_025335 transmembrane protein 167

NM_010110 ephrin B1

NM_024194 leucine rich repeat containing 40

NM_019388 CD86 antigen

NM_025376 RIKEN cDNA 1110002H13 gene

NM_207244 Cd200 receptor 2; CD200 receptor 4; RIKEN cDNA F630003A18 gene

NM_025982 tetraspanin 31

NM_206974 potassium channel regulator

NM_199366 predicted gene 9994; galactose-3-O-sulfotransferase 2

NM_025655 transmembrane and immunoglobulin domain containing 1

NM_028523 discoidin, CUB and LCCL domain containing 2

NM_022721, NM_001042659 frizzled homolog 5 (Drosophila)

NM_178669 clarin 3

NM_130448 protocadherin 18

NM_172671 leucine-rich repeat-containing G protein-coupled receptor 4

NM_026243 mannosyl (alpha-1,3-)-glycoprotein beta-1,4-N-acetylglucosaminyltransferase, isozyme C (putative)

NM_025944 RIKEN cDNA 2810432L12 gene

NM_011780 a disintegrin and metallopeptidase domain 23; similar to ADAM23

NM_030699 netrin G1

NM_010795 mannoside acetylglucosaminyltransferase 3

NM_033552 solute carrier family 4, sodium bicarbonate cotransporter-like, member 10

NM_172509 potassium channel tetramerisation domain containing 7

NM_001039195 glutamate receptor, ionotropic, AMPA2 (alpha 2)

NM_011342 SEC22 vesicle trafficking protein homolog B (S. cerevisiae)

NM_146017 gamma-aminobutyric acid (GABA) A receptor, pi

NM_172653 solute carrier family 39 (zinc transporter), member 10

NM_001039089, NM_011344 sel-1 suppressor of lin-12-like (C. elegans)

NM_019423 elongation of very long chain fatty acids (FEN1/Elo2, SUR4/Elo3, yeast)-like 2

NM_021889 synaptotagmin IX

NM_025883, NM_001033573 predicted gene 5509; zinc finger, DHHC domain containing 6

NM_175417 RIKEN cDNA 9530008L14 gene

NM_011627 trophoblast glycoprotein

NM_134110 potassium voltage-gated channel, Isk-related subfamily, gene 2

NM_008165 glutamate receptor, ionotropic, AMPA1 (alpha 1); similar to Glutamate receptor, ionotropic, AMPA1 (alpha 1)

NM_182959 solute carrier family 17 (sodium-dependent inorganic phosphate cotransporter), member 8

NM_021406 triggering receptor expressed on myeloid cells 1

NM_198295 thioredoxin-related transmembrane protein 3

NM_020278 leucine-rich repeat LGI family, member 1; predicted gene 3888

NM_021610 glycoprotein A33 (transmembrane)

NM_026164 patatin-like phospholipase domain containing 8

NM_138677 ER degradation enhancer, mannosidase alpha-like 1

NM_145511, NM_001077237 similar to odorant response abnormal 4; cDNA sequence BC003331

NM_029561 Nedd4 family interacting protein 2

NM_199200 family with sequence similarity 171, member A2

NM_019758 mitochondrial carrier homolog 2 (C. elegans); predicted gene, 100039384; predicted gene, 100039506

NM_018814 pecanex homolog (Drosophila)

NM_031186 N-deacetylase/N-sulfotransferase (heparan glucosaminyl) 3

NM_176835 DnaJ (Hsp40) homolog, subfamily C, member 22

NM_001081231 lipoma HMGIC fusion partner-like 3

NM_026281 transmembrane 7 superfamily member 3

NM_027878 RIKEN cDNA 1200002N14 gene

NM_026178 monocyte to macrophage differentiation-associated; similar to monocyte to macrophage differentiation-associated

NM_177794 transmembrane protein 26

NM_024217 CKLF-like MARVEL transmembrane domain containing 3

NM_198037 cache domain containing 1; similar to Cache domain containing 1

NM_001039560 glucosaminyl (N-acetyl) transferase family member 7

NM_172537 sema domain, transmembrane domain (TM), and cytoplasmic domain, (semaphorin) 6D

NM_026170 endoplasmic reticulum-golgi intermediate compartment (ERGIC) 1

NM_201351 cytochrome b, ascorbate dependent 3

NM_178396 carbonic anyhydrase 12

NM_016916 predicted gene 3852; bladder cancer associated protein homolog (human)

NM_023116 calcium channel, voltage-dependent, beta 2 subunit

NM_130905 CD209e antigen

NM_177766 solute carrier family 35, member E1

NM_172050 CD300e antigen

NM_172694 multiple EGF-like-domains 9

NM_001038701 gamma-aminobutyric acid (GABA) A receptor, subunit beta 3

NM_021433 syntaxin 6

NM_001033258 DNA segment, Chr 10, Brigham & Women's Genetics 1379 expressed

NM_008882 plexin A2

NM_008425 potassium inwardly-rectifying channel, subfamily J, member 2

NM_172396 RIKEN cDNA 9130011J15 gene

NM_008938 peripherin 2

NM_027133 limb and neural patterns

NM_172633 cerebellin 2 precursor protein

NM_001033439 leucine-rich repeats and calponin homology (CH) domain containing 1

NM_145561 similar to airway trypsin-like protease; transmembrane protease, serine 11d

NM_009915 chemokine (C-C motif) receptor 2

NM_028975 transmembrane protein 33

NM_148941 elongation of very long chain fatty acids (FEN1/Elo2, SUR4/Elo3, yeast)-like 4

NM_018829 adaptor-related protein complex 3, mu 1 subunit; similar to Adapter-related protein complex 3 mu 1 subunit (Mu-adaptin 3A) (AP-3 adapter complex mu3A subunit)

NM_148929, NM_178371 solute carrier family 9 (sodium/hydrogen exchanger), member 8

NM_175403 malectin

NM_028264 transmembrane protein 55A

NM_172606 membrane-associated ring finger (C3HC4) 6

NM_011334 chloride channel 4-2

NM_009194 similar to solute carrier family 12, member 2; solute carrier family 12, member 2

NM_173388 solute carrier family 43, member 2

NM_001013390 sodium channel, type IV, beta

NM_175696 RIKEN cDNA C530028O21 gene

NM_028973 leucine rich repeat containing 15

NM_145512 SFT2 domain containing 2

NM_020588 transmembrane protein 183A

NM_013933 vesicle-associated membrane protein, associated protein A

NM_021275 potassium voltage-gated channel, shaker-related subfamily, member 4

NM_025436 sterol-C4-methyl oxidase-like

NM_001013028 expressed sequence AI597468

NM_178915 hypothetical protein LOC100047659; transmembrane protein 179

NM_023122 glycoprotein m6b

NM_011348 sema domain, immunoglobulin domain (Ig), short basic domain, secreted, (semaphorin) 3E; hypothetical protein LOC100044162

NM_024290 tumor necrosis factor receptor superfamily, member 23

NM_016862 vesicle transport through interaction with t-SNAREs homolog 1A (yeast)

NM_001081320 cytochrome b-561 domain containing 1

NM_130450 predicted gene 11295; ELOVL family member 6, elongation of long chain fatty acids (yeast)

NM_028766 transmembrane protein 43

NM_176942 gamma-aminobutyric acid (GABA) A receptor, subunit alpha 5

NM_133704 SEC22 vesicle trafficking protein homologe A (S. cerevisiae)

NM_027652 DNA segment, Chr 5, Wayne State University 178, expressed

NM_153054 solute carrier family 18 (vesicular monoamine), member 1

NM_001034868 MAS-related GPR, member X2

NM_021305 Sec61, alpha subunit 2 (S. cerevisiae)

NM_029626 glycosyltransferase 8 domain containing 1

NM_177129 contactin 2

NM_026166 RIKEN cDNA 1200009F10 gene

NM_033560 vacuolar protein sorting 37A (yeast); similar to Vps37a protein

NM_009613 a disintegrin and metallopeptidase domain 11

NM_029777 rhomboid domain containing 1

NM_138754 CDP-diacylglycerol--inositol 3-phosphatidyltransferase (phosphatidylinositol synthase)

NM_025657 leucine rich repeat containing 57

NM_175414 tetraspanin 9

**Gene Group 26 Enrichment Score: 0.016578218517536866**

**GENBANK_ACCESSION Gene Name**

NM_009411 trophoblast specific protein alpha

NM_015814 dickkopf homolog 3 (Xenopus laevis)

NM_022984 resistin

NM_177139 LY6/PLAUR domain containing 6

NM_178612 canopy 4 homolog (zebrafish)

NM_001001738 inositol 1,4,5-triphosphate receptor interacting protein

NM_008752 neurexophilin 2
